# Selecting variants of unknown significance through network-based gene-association significantly improves risk prediction for disease-control cohorts

**DOI:** 10.1038/s41598-019-39796-w

**Published:** 2019-03-01

**Authors:** Anastasis Oulas, George Minadakis, Margarita Zachariou, George M. Spyrou

**Affiliations:** 1 The Cyprus Institute of Neurology & Genetics, Bioinformatics Group, 6 International Airport Avenue, 2370 Nicosia, Cyprus, P.O.Box 23462, 1683 Nicosia, Cyprus; 2 The Cyprus School of Molecular Medicine, 6 International Airport Avenue, 2370 Nicosia, Cyprus, P.O.Box 23462, 1683 Nicosia, Cyprus

**Keywords:** Computational biology and bioinformatics, Risk factors

## Abstract

Variants of unknown/uncertain significance (VUS) pose a huge dilemma in current genetic variation screening methods and genetic counselling. Driven by methods of next generation sequencing (NGS) such as whole exome sequencing (WES), a plethora of VUS are being detected in research laboratories as well as in the health sector. Motivated by this overabundance of VUS, we propose a novel computational methodology, termed VariantClassifier (VarClass), which utilizes gene-association networks and polygenic risk prediction models to shed light into this grey area of genetic variation in association with disease. VarClass has been evaluated using numerous validation steps and proves to be very successful in assigning significance to VUS in association with specific diseases of interest. Notably, using VUS that are deemed significant by VarClass, we improved risk prediction accuracy in four large case-studies involving disease-control cohorts from GWAS as well as WES, when compared to traditional odds ratio analysis. Biological interpretation of selected high scoring VUS revealed interesting biological themes relevant to the diseases under investigation. VarClass is available as a standalone tool for large-scale data analyses, as well as a web-server with additional functionalities through a user-friendly graphical interface.

## Introduction

Human genetic variation analysis has recently been influenced by next generation sequencing (NGS) technologies in the form of whole-genome sequencing (WGS), whole-exome sequencing (WES) and multigene panels. These methodologies, as well as genome wide association studies (GWAS), have paved the way for analysing genetic variation by means of global, high-throughput methods. Currently there is a growing trend to incorporate these technologies in clinical diagnosis, prognosis and even use them in guiding the choice of underlying treatment, as well as overall patient counselling. The notion of utilizing these methods regularly in medical care poses multiple challenges, one of which is how to address variants of unknown/uncertain significance (VUS)^1–5^. These VUS, such as single nucleotide polymorphisms (SNPs) or insertions/deletions (INDELs), cause dilemmas for clinicians and uncertainty on how to advise patients. Situations are further complicated when addressing complex diseases like cardiomyopathies or certain neurological disorders, because multiple genetic factors have to be taken in consideration in order to provide a more concise genetic profile for a specific patient.

Human genetic variation screening is currently focussed towards detecting a pathogenic variant in targeted high-risk individuals with an increased likelihood for a specific inheritable disease, as suggested by their family history. This approach is mainly suited to hereditary diseases such as inherited forms of breast and ovarian cancers, or certain types of “monogenic” disorders such as certain types of ataxias. This limited application of genetic testing, which relies on detection of “single” genetic variants which are “pro-disease”, supports the notion that identifying a genetic variant represents a “negative” indicator regarding health and longevity. However, this is not always the case. In order to adequately address the association of genetic variants to mortality risk and attain a complete representation of a genetic profile, it is necessary to take into consideration both the significance of variants acting in synergy towards pathogenicity as well as the “protective genetic variants”. Variant synergy can be defined as the phenomenon where a combination of variants (either pro-disease or protective) provides additive value greater than the sum of individual variants^6^. A protective genetic variant is a genetic variant associated with decreased risk of disease. Recent reviews and research on the study of protective genetic variants conclude that they are largely neglected within genomics^7,8^.

Widely accepted methods for variant analysis are based on odds ratio analysis such as those performed in traditional GWAS studies in order to obtain associations between specific, “single” variants and a disease of interest. One drawback of this methodology is that it relies on strict thresholds, namely, those variants that fall below odds ratio thresholds (e.g. <1.5) are not deemed significant due to the non-synergistic nature of traditional odds ratio calculations. This creates the “grey” zone of odds ratio analysis comprised of variants that fall slightly below thresholds and which are subsequently ignored. However, these variants may provide valuable information for disease if their mode of action is considered in a synergistic manner. Odds ratio analysis further denotes that if a variant is associated with a disease then its derived allele is disease-associated either with increased risk (being more frequent in cases than controls) or with decreased risk (being more frequent in controls than cases). In the latter case, if the variant is functionally linked to the disease (and it is not just a proxy) then it may be termed as a protective variant. However, synergies between pro-disease and protective variants are not considered in this framework. Odds ratio analysis is often followed by polygenic risk score prediction performed by pooling highly significant variants and assessing risk model performance on patient-control cohorts^9,10^.

Other methods for variant analysis include standard approaches in annotation of VUS such as those provided by *in-silico* frameworks like GEMINI^11^, whereby each variant is automatically annotated by comparing it to several genomic annotation sources such as ENCODE tracks^12^, UCSC tracks^13^, OMIM^14^, dbSNP^15^, KEGG^16^, and HPRD^17^. Moreover, allelic frequency information is provided from population genetics databases such as ExAC^18^, ESP^19^ and 1000GP^20^ (rare variants show < 1% frequency in human populations). In addition, pathogenicity related information and impact of individual variants in the context of disease is made available from databases like ClinVar^21^, COSMIC^22^ and scoring schemes like CADD^23^, Polyphen^24^ and SIFT^25^ which score the impact of the amino acid change caused by the genetic variation at the protein level.

These approaches notably, have led to important discoveries and breakthroughs in the past with success stories like the use of BRCA1/2 high penetrance genes for breast cancer genetic tests and risk assessment^26–29^. However, these methods have obvious drawbacks in the sense that they are mainly suited for detection of monogenic variants (SNPs or INDELs) associated with the disease at hand. Therefore, while the use of individual variants may provide valuable genetic information for rare monogenic disease and perhaps allow for the detection of major genetic factors for some polygenic diseases, the fact of the matter is that they fall short in capturing the full genetic profile of complex diseases.

Risk models that accurately predict disease outcome using genetic information often perform poorly when individual variants are utilized^30^. In contrast, discovering unique subsets of biologically meaningful, disease specific variants that act synergistically, may not only boost the prediction accuracy of risk models, but also provide invaluable information on the molecular basis and mechanisms of the disease. Current approaches for predicting variants with a synergistic effect on disease outcome include k-way interaction information^31^, epistasis^32,33^ or SNP synergy^34^. Other approaches use non-biologically and non-clinically informed searches to discover sets of best-interacting variants, using mutual information and information gain for individual and pairs of variants and synergy of pairs of variants^35^. These approaches are extremely time consuming as they require exhaustive searches between all possible combinations of variant pairs to detect informative variants and furthermore, they do not assess triplets or even larger groups of synergistic variants. A recent study makes use of biological evidence-based networks to narrow down their search space^6^. However, it is limited to detection of pairs of synergistic variants and does not follow-up to consider groups of multiple synergistic variants.

In this work, we propose a novel computational framework, called VariantClassifier (VarClass), which selects informative variants of unknown significance through network-based gene-association and utilizes these variants to significantly improve risk prediction for disease-control cohorts. The *VarClass* methodology (1) provides new associations between variants (previously characterized as VUS) and disease, (2) allows for detection of synergistically acting variants thus providing a more realistic view of genetic variability in complex diseases, (3) boosts the risk prediction accuracy for disease-control genetic variation cohorts by selecting informative VUS from the “grey” zone of odds ratio analysis, and (4) predicts both “pro-disease” and “protective” variants and incorporates these into its analysis pipeline allowing for the depiction of more complete genetic profile for individual patients.

## Results

### Setting up the VarClass Pipeline

**Figure 1:**
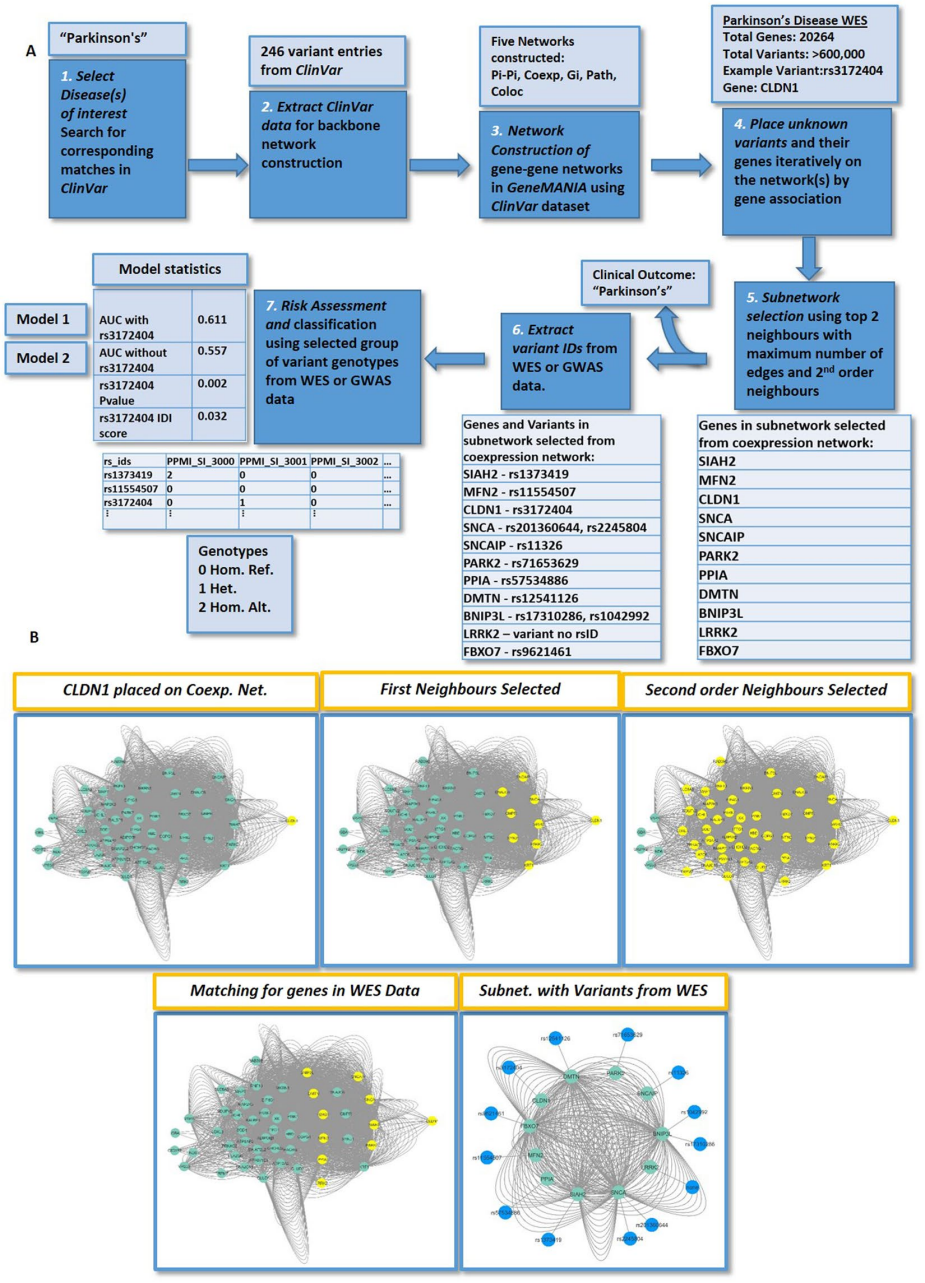
VarClass Methodology Flowchart. *Step1 - Selecting disease direction/profile* – The VarClass approach requires a general disease direction to initiate the pipeline (e.g. Parkinson’s). *Step 2* - *Extracting relevant information from ClinVar* – The relevant information by simple SQL querying, is extracted from ClinVar. *Step 3 - Network Construction* – The gene information (gene symbols) extracted from all entries associated with the disease profile (as defined in steps 1 and 2) are used to construct the backbone of five different types of gene-to-gene networks using GeneMANIA. *Step 4 - Placing unknown variants on the networks* – unknown variants (e.g. variant: rs3172404 in gene: CLDN1) are placed iteratively on all five networks by means of gene association. *Step 5 - Defining the sub-network of informative variants* – Firstly, this step involves the selection for the top 2 neighbours of the gene harbouring the VUS. These neighbours are next used for *prediction of clinical outcome for VUS (e.g. Parkinson’s)*. Secondly, the subnetwork is further expanded by selecting the 2^nd^ order neighbours (i.e. neighbours of the top 2 neighbouring genes), hence adding even more informative genes for the next processing steps of the analysis pipeline (these genes are shown in the first light blue table). *Step 6 - Extract variant IDs from real data* – this next step involves the use of real GWAS/WES data and adding all the variants from the GWAS/WES datasets to their corresponding genes present in the selected subnetwork(s) (genes and variants are shown in the second light blue table). *Step 7 - Using variants derived from sub-networks for risk prediction* – The variants obtained from the sub-network are used in the risk model construction using the genotypes from all disease and control samples in the GWAS/WES study (genotypes are shown in the third light blue table). Two types of risk models are generated. Namely, Model 1 - which contains all the sample genotypes from the variants found in the subnetwork and Model 2 - a second model that contains all genotypes *without* the genotypes of the VUS that is under investigation at that given iteration. The difference in AUC, NRI and IDI between the two models provides a means of assessing the contribution of the VUS under investigation (model statistics are shown in the fourth light blue table). (**B**) Details on subnetwork selection process (*Step 5*) using specific example from Parkinson’s WES. The green nodes represent genes found in gene-gene co-expression network, which achieves significant results for this specific variant iteration. The yellow nodes represent the gene/variant been analysed in this VarClass iteration as well as the accompanying selected genes that ultimately make up the synergistic group in the final subnetwork. The selection process entails a 2 stage process, first neighbours with maximum number of edges are selected and then the second order neighbours of these genes are also selected. Finally, the genes/nodes with available genotype information from the WES data are selected to construct the final subnetwork for downstream risk assessment analysis.

We initially set up the VarClass Pipeline by implementing a series of components (as illustrated in Fig. 1). For the first 2 steps in the pipeline, ClinVar (https://www.ncbi.nlm.nih.gov/clinvar/)^21^ is used to extract known variants and genes associated with a disease direction of interest. ClinVar database is a publically accessible repository encapsulating relationships between human variations and phenotypes, observed health status and clinically relevant information for genes harbouring variations. This information is concurrently used to infer clinical disease-related information for variants of unknown significance via gene association. This is achieved in the next 2 steps, which entail the construction of five different types of biological, evidence-based, backbone networks using GeneMANIA^36^. These include protein-protein interaction, co-expression, co-localization, genetic interaction and common pathways networks. Variants of unknown significance (VUS) from large-scale data (i.e. WGS, WES, multigene panels or GWAS) are then placed on these networks iteratively by gene association (steps 3–4). Steps 5 and 6 describe the selection of an informative subnetwork by selecting neighbouring nodes of the variant gene and extracting variants from large-scale data for all genes in the subnetwork. Finally, VarClass makes use of these variants derived from the network-based gene association information and couples it with a polygenic risk model approach to detect groups (> = 2) of synergistically acting variants required to accurately predict disease outcome. This final step of the analysis flow chart (step 7) generates 2 types of Risk models: Model 1 - contains all the sample genotypes from the variants found in the subnetwork and Model 2 - a second model that contains all genotypes except for the genotype of the VUS that is under investigation at that given iteration. Risk prediction is performed using both Models 1 and 2 and differences assessed using receiver operating characteristic (ROC) curve, area under the curve (AUC) and Integrated Discrimination Improvement (IDI)^37^ measures. Thus, providing a significance score for the specific variant. The IDI is a measure of improvement in model performance and quantifies how well a new model reclassifies the data.

### Validation of VarClass

The validation strategy adopted here involves the use of two panel array datasets. These are datasets characterized by a relatively small number of targeted, focussed variants specific to a disease. In addition, we also included mock-generated data in order to validate three specific aspects of our methodology: (1) the use of VarClass to provide an informative ranking score to pro-disease as well as protective variants, (2) the capacity of VarClass to predict synergies between variants that show greater significance as a group in comparison to assessing individual variants alone, and (3) the ability of VarClass to classify VUS to a specific clinical outcome by placing them in gene-to-gene disease specific networks.

#### Validation of VarClass in informative ranking of pro-disease and protective variants

The power of VarClass to provide an informative ranking score to pro-disease as well as protective variants is evaluated by using the two panel array datasets: GSE8055 (141 pancreatic cancer, 89 controls patients) and GSE8054 (121 pancreatic cancer cases, 87 controls). These panels contain documented cancer variants that have previously been used in studies for pancreatic cancer genetic variation^38^.

We used all variants present in the GSE8055 (n = 928) and GSE8054 (n = 1189) studies to generate genetic risk prediction models. We then assessed the performance on the data using a leave-one-out cross validation procedure. Risk prediction for both datasets achieve a very high prediction accuracy as shown by ROC curves with AUC scores of 0.999 and 0.996 for GSE8055 and GSE8054 respectively (see Supplementary Fig. S1). The high prediction accuracy of these results is expected as the genes and variants present in these panel datasets are known to be implicated in cancer.

The above results show that the variant genotypes from these datasets are highly informative in discriminating between disease and control samples. To simulate uninformative (true negatives) variants we used an imputation algorithm (R package linkim^39^) to compute the proportions of the different genotypes in each patient of the GSE8055 datasets and generate additional (~1600) imputed variants that closely resemble the true patient genotype distributions. After pooling the true positive and true negative (or “mock”) variants into one dataset, we used it as input for the VarClass pipeline selecting a general disease direction with keywords “Pancreatic Cancer” and including “adenocarcinoma” and “Colon Cancer”. The same procedure was repeated for the GSE8054 dataset.

**Figure 2:**
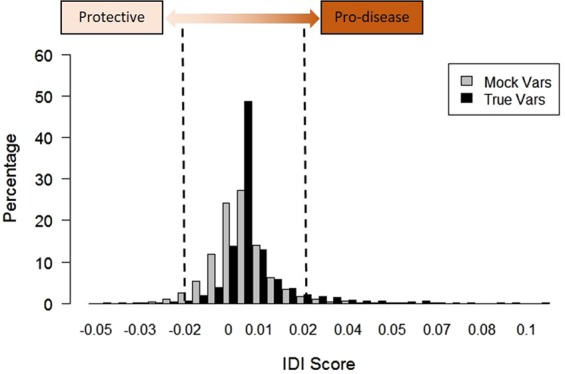
VarClass Score for Selecting Pro-disease and Protective Variants. VarClass output showing True versus Mock (imputed) variants distribution using IDI score for the validation cohorts GSE8055 (n = 928) and GSE8054 (n = 1189), known to be associated with Pancreatic Cancer. T-test results: t = 13.345 for df = 3212.6, and a p-value: < 2.2e-16. The brown gradient arrow and dashed lines show the selected cut-offs of 0.02 and −0.02 for pro-disease and protective variants respectively.

Using these datasets as input to VarClass, we attain top scoring synergistic variants showing high accuracy in risk prediction. In order to assess the significance between true informative variants and the imputed “mock” variants VarClass generates two Models (1 and 2) and assesses risk prediction accuracy by including or excluding the variant under investigation respectively. VarClass uses the IDI score to measure improvement in model performance and quantify how well a new model reclassifies the data^37^. We show that the distributions of IDI scores obtained from the true informative variants and those derived from the imputed “mock” variants differ significantly. T-test results (t: 13.345, degrees of freedom (df): 3212.6, p-value: < 2.2e-16) favour the alternative hypothesis that true difference in means is not equal to zero. In order to select a cut-off IDI value for selecting informative variants we looked at the cut-off achieving >95% specificity for both variant types, thus minimizing false positive rate. This IDI score cut-off was at 0.02 and −0.02 for pro-disease and protective variants respectively (see Fig. 2). This validation step provides proof of concept that utilizing the VarClass pipeline; it is possible to attain informative variants for a specific disease of interest. In essence, by treating these panel dataset variants, together with mock variants, as “unknowns” we show that it is possible to re-capture the information attained from association studies via network methodologies and furthermore allows for a prediction of pro-disease as well as protective variants.

#### Validation of VarClass in selecting informative variants acting in synergy

The concept of variant synergy is that combination of variants provides greater additive value than individual variants. With this in mind, we further performed an additional form of validation for our approach to show the ability of VarClass to detect synergies and assess the impact of synergistic informative variants on the AUC and IDI scores generated by VarClass. This is achieved by extracting the top scoring informative VarClass variants as well as their additional synergistic partner variants. The synergistic partners are variants selected from neighbouring nodes of the network backbone as described in VarClass pipeline Fig. 1 steps 5–7. We next performed incremental removal of all synergistic partners in these informative synergistic groups and re-classified the validation datasets using risk prediction models. This evaluation showed that significant AUC and IDI differences between Models 1 and 2 could only be accounted for upon removal of the top scoring variants. An example of this scenario is the rs6905948 variant (IDI 0.091, p-value: 0.059), in the PTK7 (Protein Tyrosine Kinase 7) gene, known to be implicated in colon carcinoma as well as in some types of anxiety-related disorders (see Supplementary Fig. S2). This validation step shows that individual VarClass top scoring variants are highly informative to their synergistic group with respect to risk prediction for disease-control cohorts and their removal causes a significant decline in risk prediction accuracies.

In addition to the above step that shows the impact of removal of individual variants from the synergistic group, we further assessed the risk prediction performance utilizing individual top scoring variants alone. Individual top scoring variants were used to generate risk models, results showed that the use of single variants generated very poor models with low performance and AUC values ranging from 0.500–0.644. An example of using top scoring variant rs6905948 alone to perform risk prediction, generated a leave-one-out cross validation prediction accuracy of 63.04%, a sensitivity of 76.60%, a specificity of 41.57% and a Matthews Correlation Coefficient of 0.19.

Overall the combination of these validation procedures show that VarClass obtains highly significant synergistic groups of variants with top scoring variants having detrimental effects on risk prediction upon removal. Moreover, these top scoring variants fail to achieve high prediction accuracies when utilized alone in risk prediction models.

#### Validation of VarClass in assigning VUS to a specific clinical outcome

We validated the capability of VarClass to assign VUS to a certain clinical outcome by compiling a validation dataset of neurological related diseases from ClinVar. This dataset included Alzheimer’s (AD), Parkinson’s (PD), and other dementia-related diseases. The dataset comprised of unique entries for 22 disorders, 70 genes and 385 variant IDs. We performed a cross-validation analysis by splitting the dataset equally into training and test set and assessing the classification performance of VarClass on the test set. Results are averaged over 100 cross-validation runs for the neurological disease validation dataset. Specifically for every run, network construction is performed (as described in step 3 of the VarClass pipeline) using both test and training sets. The “unknown” nodes (from the test set) are subsequently classified according to their neighbouring “known” nodes (from the training set). The neighbours of each “unknown” node are ranked according to the highest number of interactions (edges) they have with this node. Concurrently the “unknown” nodes (gene and associated variant) are assigned to the same clinical outcome category as its closest neighbours. This creates a small but highly informative sub-network suitable to extract useful clinical information.

After testing for different values of *k* neighbours of the “unknown” node, optimal results are achieved by selecting the top two neighbouring nodes of the test gene under classification. The prediction accuracy was 76.64% in correct disease prediction averaged over the 100 cross-validation runs. This validation approach provides support that information gained by gene association from biological evidence-based, disease specific networks, can provide additive value towards predicting a clinical outcome for VUS.

### Application of VarClass using genetic variation datasets as case studies

The application of VarClass was assessed using four datasets from three different diseases/disorders as case studies. These are two intellectual disability datasets, one gastric cancer dataset and one PD dataset (see Supplementary Table S1). Initially we performed standard odds ratio analysis on all of these datasets. This provides a set of variants associated with disease or protective against disease, which are concurrently used to create a risk prediction model. The risk prediction accuracy obtained by this model for diseases vs. control samples sets the baseline for assessing the performance of VarClass. Overall, the application of VarClass (1) achieved selection of a set of top scoring informative variants, (2) boosted risk prediction accuracy for all case studies by including top scoring variants to the baseline model generated by odds ratio analysis and (3) proved capable of selecting biological significance top scoring variants in the context of the diseases under consideration (details below).

#### Setting a baseline for VarClass using odds ratio analysis

Odds ratio analysis is performed on all datasets and an odds ratio threshold is selected depending on the dataset in hand. We tried various different values (in an incremental manner) for setting the baseline threshold using odds ratio analysis (data not shown) and the thresholds that generated the optimal results are reported here. For the PD dataset an odds ratio >5 attained 560 variants all of which are high risk penetrant variants (no protective). Results from training a polygenic risk model using these 560 variants obtained high prediction accuracy and an AUC value of 0.8 according to leave-one-out cross validation analysis. Similarly for the gastric cancer dataset an odds ratio of >5 attained 152 variants, most of which were high risk penetrant (124/165) and some protective (41/165). Results from training a polygenic risk model using the following variants obtained high prediction accuracy and an AUC value of 0.725 according to leave-one-out cross validation. Interesting results are obtained when performing odds ratio analysis for the intellectual disability dataset GSE7226 Platform: GPL2005. The optimal threshold was set at 1.5, however, most of the variants exceeding this odds ratio appeared to be protective (724/833). These variants are more frequently occurring in control samples compared to affected samples and as shown later by our risk prediction models, they are associated with decreased risk of disease for the affected individuals. In order to accurately view these variants in ROC curves the classes are reversed and thereafter showing an AUC value of 0.796. Similar results are obtained for the GSE7226 Platform: GPL2004 intellectual disability dataset where variants attaining high odds ratio values showed protective characteristics. Risk prediction accuracy for this dataset selecting for variants with odds ratio >2 attained an AUC value of 0.733.

The above mentioned analysis, including the ROC-generated AUC scores, is used as a baseline for evaluating the high scoring variants selected by the VarClass methodology.

#### Selecting informative variants using VarClass

Running the VarClass pipeline on the four large-scale datasets described above allowed ranking of variants based on synergy and impact on risk prediction. As confirmed in the validation steps, VarClass capitalizes on biologically meaningful, disease specific networks to select subsets of variants acting in synergy. These variants are used to predict disease risk and delineate disease outcome. The VarClass pipeline is initiated by selecting the relevant disease direction for each of these datasets and extracting the relevant information from ClinVar. Next, backbone networks are generated and individual variants are placed on these networks by gene association. This step is followed by sub-network selection and variant assessment using risk models. At first glance, our results reveal differences in the prediction accuracy attained by VarClass risk models when assessing individual variants, with some models showing high accuracy and some low. Interesting results are observed when we performed integration of different types of biological networks. For each variant under assessment by our approach, we choose to integrate biological networks that achieved significant results (e.g. co-expression with genetic interaction networks). Integration is performed by merging these networks together by common gene(s) and pursuing our analysis in a similar manner as for a single network. This time the networks are enriched with multi-level information and the selected neighbours are also enriched with additional biologically informative genes. Consequently, this leads to the inclusion of additional informative variants in our risk model training/classification step and an overall improvement of VarClass performance.

Following completion of the VarClass pipeline for each dataset we obtained a scoring scheme for each variant in the assessed datasets. Variants can be sorted according to different scores (e.g. coefficient p-value, see Supplementary Tables S2–S5). We choose the IDI score as it is considered to be one of the most reliable measures of assessing model performance during classification procedures. For each dataset we record the number of unique genes present in the data, the total number or risk models generated by VarClass, the number of risk models with IDI score < = −0.02 for protective variants and > = 0.02 for pro-disease variants and finally the number of top scoring VarClass variants. Each model represents a single variant; however, there may be more than one model per variant depending on the network used to generate the model. (e.g. co-expression and protein-protein interaction networks)

Overall, the application of VarClass was successful in obtaining informative variants for the four case studies. A summary of results per dataset is shown in Table 1. Detailed information on genes and selected variants per dataset can be viewed in Supplementary Tables S2–S5.

**Table 1:** Summary Results of top scoring Variant selection by VarClass per dataset.

Dataset	Disease^a^	Unique genes	Risk Models^b^	Protective Models (IDI score < = −0.02)^c^	Pro-disease Models (IDI score > = 0.02)^c^	No. of top scoring VarClass Variants^c^
GSE7226 Platform: GPL2005	Intellectual disability	2486	16551	82		82
GSE7226 Platform: GPL2004	Intellectual disability	2676	18992	51		51
GSE58356	Gastric cancer	9256	15272		12	10
PPMI	Parkinson’s	20264	6253		25	22

^a^Disease keywords are used in stages 1 and 2 of the VarClass flow Chart (see Fig. 1) to initiate the disease profile and extract information from ClinVar.

bRisk Models are derived from stages 3–8 of the VarClass pipeline, where each model represents a different type of network used to obtain information of VUS by gene association.

^c^Protective and Pro-disease models derived after filtering the results from step 7 (final outcome) of VarClass by applying IDI Thresholds and selecting a final number of Protecting and Pro-disease models and their associated variants.

#### Improvement of Risk prediction using VarClass Variants

Application of VarClass allowed the selection of a specific set of pro-disease (IDI > = 0.02) or protective variants (IDI < = −0.02) for each of the four datasets. Consequently, these variants, initially considered as VUS, are added to the baseline odds ratio variants for each dataset. Leave-one-out cross validation is performed in order to assess the risk prediction accuracy before and after inclusion of these top scoring VarClass variants to the baseline odds ratio variants. ROC curves, box plots and distribution plots are obtained for all four datasets.

**Figure 3:**
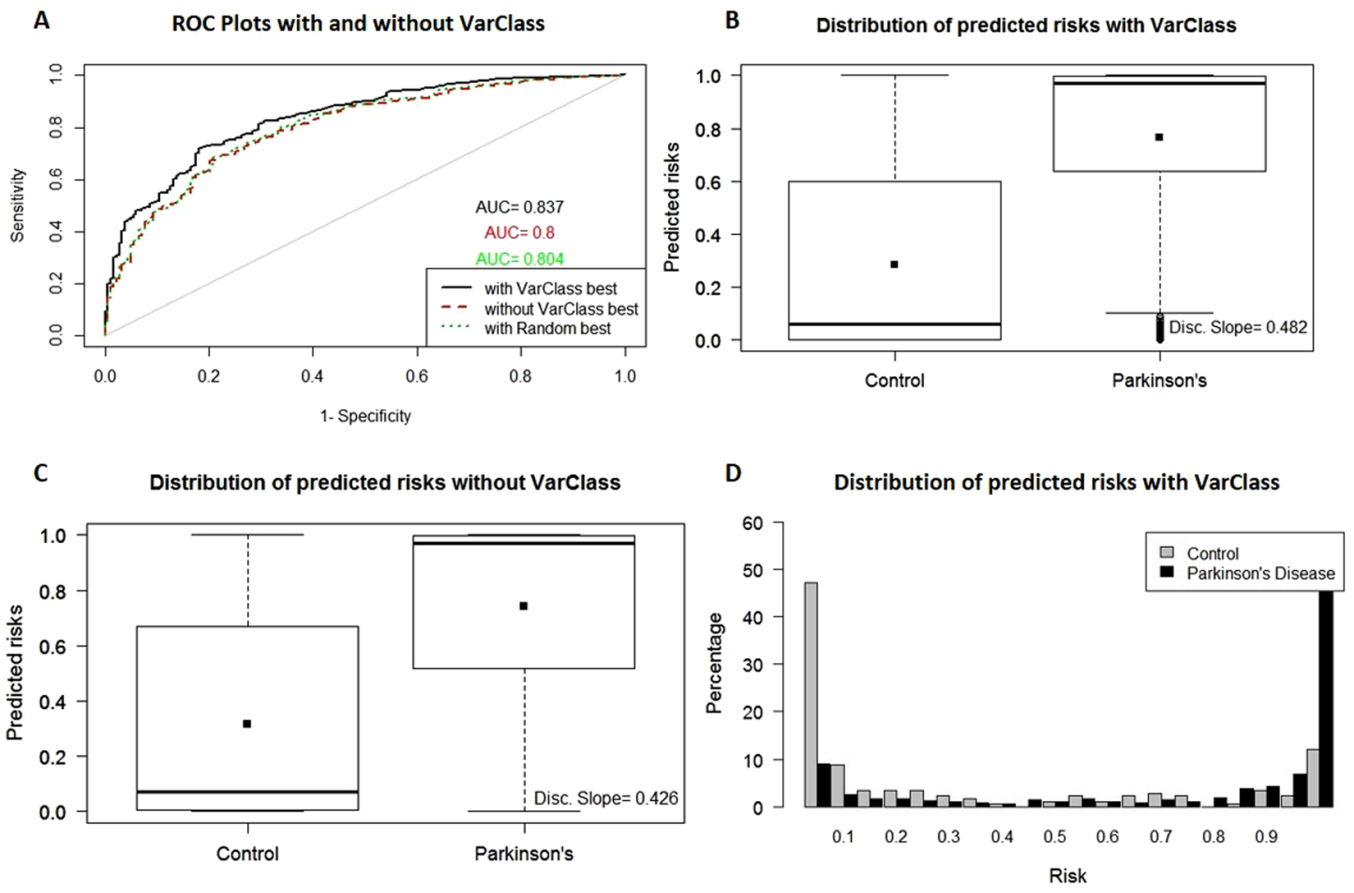
VarClass improvement of Risk Production for Parkinson’s dataset. (**A**) ROC curve showing classification of Parkinson’s disease and normal samples. Black and red lines denote logistic binomial regression classification when including and excluding informative VarClass variants. The green dotted line shows prediction accuracy from including random variants to the baseline odds ratio variants for this dataset (**B**) Boxplot showing predicted risk mean and standard deviation for disease and control samples when including VarClass variants in the analysis. (**C**) Boxplot showing predicted risk mean and standard deviation for disease and control samples *without* including VarClass variants in the analysis. Boxplots discrimination slopes (Disc. Slope - difference between means of disease and normal populations) show a greater discrimination capacity between disease and normal samples when VarClass variants are included in the risk prediction model (0.482) and a drop in discrimination slope (0.426) when excluding the variants from the model. (**D**) The risk score distribution statistics for disease (black histogram) and control (grey histogram) including VarClass variants in the analysis.

Parkinson’s Dataset - ROC curves show a significant improvement (p-value: 3.1e-4) in risk prediction for the PPMI dataset after comparing the baseline AUC (0.8) from odds ratio analysis and VarClass AUC (0.837) after including top variants (IDI > = 0.02) in the polygenic risk score prediction (Fig. 3A). The additive value of VarClass variants in risk prediction assessment for PD patients versus controls, can also be observed in boxplot discrimination slopes and distribution plots (Fig. 3B–D). As an additional validation procedure, we used a random sampler to extract variants from the PD dataset and add them to the baseline variants. The procedure is repeated for 200 random sampling iterations and leave-one-out cross validation results are obtained by averaging predictions for every patient. ROC curves for baseline, VarClass and random sampling results are plotted together for comparison. Random addition of variants showed no significant AUC differences when compared to the baseline odds ratio analysis leave-one-out cross validation results (see Fig. 3A).

Upon closer inspection of VarClass selected variants, we find biologically significant top scoring variants for the PD dataset. These include the top scoring and highly significant variant rs3172404 (coefficient p-value: 2e-3 – obtained from linear regression analysis) with no documented entry in ClinVar found in the Claudin 1 (CLDN1) gene. This variant is used as an example case in Fig. 1 delineating all the key intermediate stages in parallel with the flowchart. CLDN1 may be a key player in PD as it is involved in tight-junction formation at the blood-brain barrier. Moreover, GTEx^40–42^ data (which describe the relationship between genetic variation and gene expression in human tissues) show notable CLDN1 RNA integrity in the brain as well as other tissue. Recent studies show a significant correlation between blood-brain barrier dysfunction and the progression of PD^43,44^. Investigating deeper into the molecular role of this variant on gene function, we find that rs3172404 is located on the boundaries of the 3’UTR and intronic region of CLDN1 and thus does not appear to affect protein synthesis of this gene. However, genomic information from the UCSC genome browser shows that in fact this region shows high splicing activity according to EST data. Disruption of splicing is known to have detrimental effects on transcriptional and hence protein synthesis and has been associated with numerous diseases^45^. Variant rs3172404 together with additional 12 variants, which make up the synergistic partners selected from neighbouring nodes of the co-expression network, show significant differences between Models 1 and 2. IDI score at the 95% confidence interval (CI) attains a value of 0.032 in favour of Model 1.

In addition to biologically informative variants and genes, pathway enrichment analysis reveals biologically significant subnetworks for the PD dataset. By selecting networks that contribute to generating the most significant risk prediction accuracy it is possible to obtain pathway information relevant to the disease of interest. Using the genes that constitute these networks, enrichment analysis can be performed allowing for insights as to the most abundant molecular mechanisms involved. For the PD dataset selecting the genes for the top variant rs3172404 described above and its synergistic partners, allows for pathway enrichment analysis using Gene Ontology (GO) database (Supplementary Fig. S3). Interesting results are observed with the most significant q value (0.0185) being attained by GO terms: GO:0044455 (mitochondrial membrane part) and GO:0031966 (mitochondrial membrane). Current thinking about PD (as well as other neurodegenerative disease) is that it is a disorder of mitochondria, the energy-producing organelles inside cells, causing neurons in the brain’s substantia nigra to die or become impaired^46–48^. As expected additional ATP/mitochondrial related processes obtained highly significant q-values. Notable examples are: GO:0007005 (mitochondrion organization, q = 0.0215), GO:0016638 (oxidoreductase activity, acting on the CH-NH2 group of donors, q = 0.0247), GO:0022904 (respiratory electron transport chain, q = 0.0247), GO:0022900 (electron transport chain, q = 0.0247) and GO:0042773 (ATP synthesis coupled electron transport, q = 0.0247).

**Figure 4:**
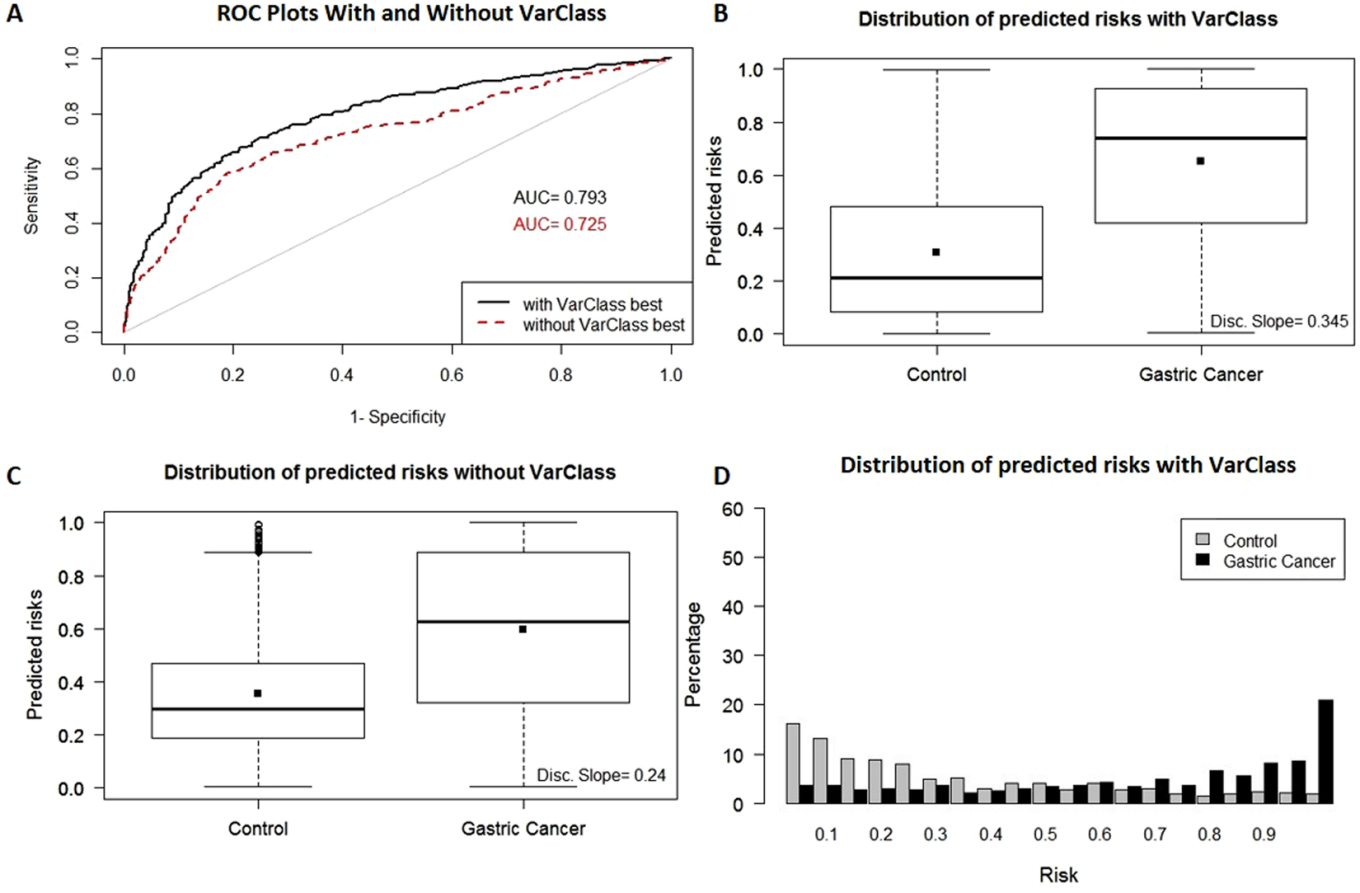
VarClass improvement of Risk Production for Gastric Cancer dataset. (**A**) ROC curve showing classification of gastric cancer and normal samples from GSE58356 dataset. Black and red lines denote logistic binomial regression classification when including and excluding informative VarClass variants. (**B**) Boxplot showing predicted risk mean and standard deviation for disease and control samples when including VarClass variants in the analysis. (**C**) Boxplot showing predicted risk mean and standard deviation for disease and control samples *without* including VarClass variants in the analysis. Discrimination slope provides a measure of quantification for the change in statistics. Showing a greater discrimination capacity between disease and normal samples when VarClass variants are included in the risk prediction model (0.345) and a drop in discrimination slope (0.24) when excluding the variants from the model. (**D**) The risk score distribution statistics for disease (black histogram) and control (grey histogram) including VarClass variants in the analysis.

Gastric Cancer Dataset - The gastric cancer dataset is used as input to the VarClass pipeline and results evaluated using the same statistical methods described for the PPMI dataset. ROC curves in Fig. 4A show risk prediction for comparing the baseline AUC (0.725) from odds ratio analysis of this dataset and VarClass AUC (0.793) after including top VarClass variants (IDI > = 0.02) in the polygenic risk score prediction. Once again, a significant improvement (p-value: 1e-5) in risk prediction accuracy is attained upon inclusion of the VarClass pro-disease variants in the cross-validation procedure. Boxplot discrimination slope values and distribution plots further highlight the improvement of risk prediction using top VarClass pro-disease variants for this dataset (Fig. 4B–D).

VarClass selected variants for the gastric cancer dataset include the biologically significant, top scoring, variant rs138618143 (coefficient p-value: 8.93e-09), also with no documented entry in ClinVar. This variant is found in the CSTF2 (Cleavage Stimulation Factor Subunit 2) gene. Expression of CSTF2 is well established in the gastrointestinal tract and has previously been associated with stomach as well as other cancers (according to the Human Protein Atlas database^49^). CSTF2 is known for its involvement in the 3’-end cleavage and polyadenylation of pre-mRNAs. Polyadenylation process has been shown to be implicated in numerous diseases including multiple cancers^50^. This specific variant is annotated as a missense variant (G [Gly] ⇒ A [Ala]), however due to its genomic location it is also designated as a splice region variant, as its sequence occurs within the region of a splice site. Variant rs138618143 together with additional 27 variants, which make up the synergistic partners selected from neighbouring nodes of the merged networks from co-expression, co-localization and genetic interaction, show notable differences between Models 1 and 2. IDI score at the 95% CI once again favours Model 1 with a value of 0.078, while attaining IDI p-value: 3.76e-04.

**Figure 5:**
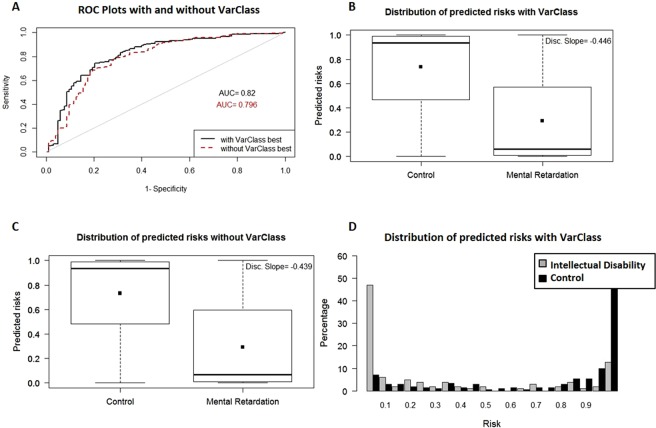
VarClass improvement of Risk Production for Intellectual Disability GSE7226-GPL2005 dataset. (**A**) ROC curve showing classification of intellectual disability and normal samples from GSE7226-GPL2005 dataset. Black and red lines denote logistic binomial regression classification when including and excluding VarClass protective variants. IDI [95% CI]: 0.0344 [0.011–0.058]; p-value: 3.5e-3. (**B**) Boxplot showing predicted risk mean and standard deviation for disease and control samples when including VarClass variants in the analysis. (**C**) Boxplot showing predicted risk mean and standard deviation for disease and control samples *without* including VarClass variants in the analysis. Discrimination slope provides a measure of quantification for the change in statistics. Showing a greater discrimination capacity between disease and normal samples when VarClass variants are included in the risk prediction model (−0.446) and a drop in discrimination slope (−0.439) when excluding the variants from the model. (**D**) The risk score distribution statistics for disease (grey histogram) and control (black histogram) including VarClass variants in the analysis.

**Figure 6:**
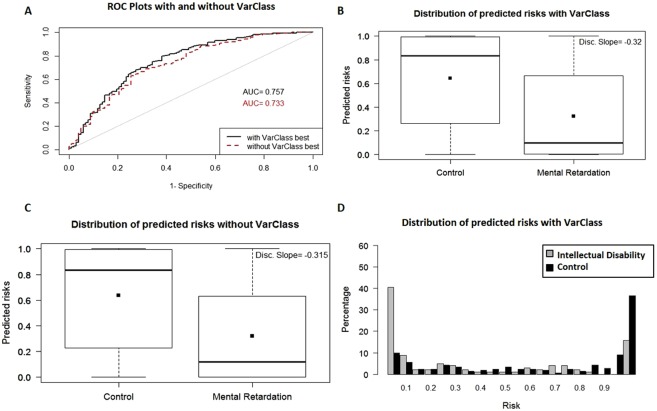
VarClass improvement of Risk Production for Intellectual Disability GSE7226-GPL2004 dataset. (**A**) ROC curve showing classification of intellectual disability and normal samples from GSE7226-GPL2004 dataset. Black and red lines denote logistic binomial regression classification when including and excluding VarClass protective variants. IDI [95% CI]: 0.0419 [−0.003–0.087]; p-value: 0.069. (**B**) Boxplot showing predicted risk mean and standard deviation for disease and control samples when including VarClass variants in the analysis. (**C**) Boxplot showing predicted risk mean and standard deviation for disease and control samples *without* including VarClass variants in the analysis. Discrimination slope provides a measure of quantification for the change in statistics. Showing a greater discrimination capacity between disease and normal samples when VarClass variants are included in the risk prediction model (−0.32) and drop in discrimination slope (−0.315) when excluding the variants from the model. (**D**) The risk score distribution statistics for disease (grey histogram) and control (black histogram) including VarClass variants in the analysis.

Intellectual Disability Datasets - The two intellectual disability datasets are characterized by protective variants. Accuracy is again improved for both datasets (GSE7226 Platform: GPL2005 and GSE7226 Platform: GPL2004) when comparing baseline AUC values (0.796, 0.733 respectively) and VarClass AUC values (0.82, 0.757 respectively) after including the top VarClass protective variants (IDI < = −0.02) in the polygenic risk score prediction (Figs 5A and 6A). There is a clear reversal in risk prediction for these datasets as shown by the boxplot and distribution plots. Both datasets show that these variants are associated with decreased risk for these patients, in accordance to the results expected from protective variants (see Figs 5B–D and 6B–D).

Investigating the intellectual disability VarClass selected variants, which are primarily characterized by protective variants, there are again interesting results of biological significance. The GSE7226 Platform: GPL2005 dataset includes the top variant rs10494979 with an apparent protective role, found in the PTPN14 (Protein Tyrosine Phosphatase, Non-Receptor Type 14) gene. The gene has previously been implicated in Choanal Atresia and Lymphedema and other types Lymphedemas and certain cancers. However, there has never been a link to intellectual disability. Although rs10494979 is an intronic variant, its genomic location is characterized by enhanced copy number gain activity. This region, stemming from chr1:187143981–224299417, has a x3 copy number gain and is deemed pathogenic according to ClinVar database. Thus, although rs10494979 may not infer obvious molecular perturbations on gene function, it may have the potential to act as reliable genetic marker for diagnosis of certain intellectual disability disorders. Variant rs10494979 together with additional 95 variants, which make up the synergistic partners selected from neighbouring nodes of the co-expression network, show highly significant differences between Models 1 and 2. IDI measure at the 95% CI attains a value of −0.037, however, this time in favour of Model 2. Results for the GSE7226 Platform: GPL2004 dataset includes the top negative scoring (protective role) variant rs10493766 found in the DDAH1 (Dimethylarginine Dimethylaminohydrolase 1) gene. The gene has previously been shown to be implicated in certain cancers (e.g. Diffuse Intrinsic Pontine Glioma), however, there has never been a link to intellectual disability. This variant is also intronic and located in a region of copy number variability. Specifically its genomic location (chr1:83457325–104273917) is characterized as x3 gain and pathogenic according to ClinVar. These observations support the possible relevance of rs10493766 as a genetic marker for diagnostic purposes. Variant rs10493766 together with additional 124 variants, which make up the synergistic partners selected from neighbouring nodes of the merged networks from protein-protein interactions, co-expression, co-localization and genetic interactions, show significant differences between Models 1 and 2. IDI score at the 95% CI attains a value −0.032 in favour of Model 2. Detailed information on biological significant variants described here can be viewed in Supplementary Tables S6–S9.

In general, the VarClass approach lead to improvement of risk prediction for all case studies assessed here, when compared to baseline risk prediction. Moreover, biologically meaningful disease-specific scenarios appear when taking a closer look at VarClass selected variants and their respective genes. A summary of the risk prediction improvement for all four datasets used as case studies for the application of VarClass is shown in Table 2.

**Table 2:** Risk prediction improvement for four datasets used to evaluate VarClass performance.

Dataset	Disease	Baseline AUC	VarClass improved AUC	IDI	p-value significance of IDI
GSE7226 Platform: GPL2005	Intellectual disability	0.796	0.82	0.0344	3.52e-3
GSE7226 Platform: GPL2004	Intellectual disability	0.733	0.757	0.0419	0.069
GSE58356	Gastric cancer	0.725	0.793	0.1054	1e-5
PPMI	Parkinson’s	0.8	0.837	0.0562	3.1e-4

## Discussion

We have developed, validated and applied VarClass, a novel computational framework suited for performing downstream analysis on genetic variation data derived from high-throughput methodologies, in order to provide a disease-related ranking score for VUS.

A discussion analysis of our results compared with the findings obtained from previous studies that analysed the same four available datasets used to test VarClass, is required at this stage. Firstly, it is important to stress that the VarClass approach is unique, in the sense that it encapsulates information obtained from biological networks and looks for variant synergies within defined network neighbourhoods. The novelty of our approach does not compare easily to traditional approaches. Nevertheless, we perform a comparison with a recent study that used traditional baseline genetic association analysis to obtain genetic risk score models for PD predictability using part of the PPMI data^51^. The authors report an AUC of ~0.74, which is markedly lower than the ~0.84 AUC achieved by VarClass for the PPMI dataset. Moreover, due to the nature of VarClass to extract variants that are otherwise not deemed significant by strict thresholds employed by other methods, the top scoring PD VarClass variants are not reported in the above mentioned study. Other published studies using the PPMI dataset included additional clinical and patient demographic data, in combination to genetic data to boost their model and report higher AUC values^52^. However, this type of data integration is not the focus of VarClass, which concentrates on boosting risk prediction accuracy using solely genetic information and specially by detecting synergistic acting variants previously designated of unknown significance. In future work, we aim to also incorporate additional features in our models, such as clinical and demographic data.

With respect to the Gastric Cancer dataset (unpublished study), we did not identify other risk prediction attempts or AUC values. However, contributors for this dataset at the GEO website (https://www.ncbi.nlm.nih.gov/geo/query/acc.cgi?acc = GSE58356) report some genetic associations of key variants, including variants of the TRIM 40 gene. This gene was also scored in the top 10 genes of informative variants by VarClass (out of 9256 unique genes assessed for this specific dataset) (see Supplementary Table S3).

The authors that analysed the intellectual disability dataset (Friedman J. M. *et. al*.^53^) performed a completely different analysis for this dataset by assessing copy number variations (CNV). This makes it difficult to compare their results with the single nucleotide variation (SNV) approach, which was employed by VarClass for this dataset. Similarly, the datasets used for validation of VarClass were previously used by Tan A. C. *et. al*.^38^ for a different type of analysis. This study assessed allele-specific expression in the germline of patients with familial pancreatic cancer and the authors do not report risk prediction accuracies in their analysis.

The results obtained for the intellectual disability datasets raise some important issue, that is, whether certain diseases or disorders (e.g. intellectual disability disorders) can be characterized by an increased number of protective variants. Furthermore, what is the role of cancer genes (DDAH1 and PTPN14) shown to have variants with a protective role for intellectual disability. Evidence from recent studies have shown that specific cancer incidence among individuals with intellectual disability may be anti-correlated or mutually exclusive^54^. More specifically, epidemiological studies for the co-occurrence of lung cancer and Down’s syndrome showed a very low rate of lung cancer incidence in these patients^55–57^. Furthermore, mortality studies reported similar findings with a significant absence of lung cancer in Down’s syndrome patients, thereby indicating that the lung cancer condition may be protective over Down’s Syndrome and vice versa^58,59^. Similarly, in fragile-X syndrome, the most common form of inherited intellectual disability, only two lung tumors have been reported^60–62^. This further supports the evidence that some types of cancers and intellectual disability may be mutually exclusive, generating the hypothesis that some cancer related genes like, DDAH1 and PTPN14, may act as protective against some forms of intellectual disability. In any case, this is a very challenging question, which, we aim to address in future work.

Overall, our results show that the VarClass methodology is a reliable method of selecting informative synergistic, pro-disease as well as protective variants from large-scale genetic variation studies. VarClass capitalizes on the grey zone of traditional odds ratio analysis and re-ranks VUS according to statistically significant measures. Moreover, VarClass analysis exploits the synergistic relationship of variants, providing a more realistic view of genetic variation for complex diseases. Importantly, validation of VarClass shows that using polygenic risk prediction to classify samples allows for individual variant evaluation using the IDI score. Inclusion of top scoring variants from VarClass in risk prediction models, shows significant improvement in risk prediction accuracy in comparison to using odds ratio variants alone, as illustrated in this work for four large scale datasets.

One of the technical limitations of VarClass is the prerequisite for a gene associated with the variant under consideration. Hence, VarClass cannot score all variant types. Specifically, some variants derived from whole genome sequencing data for example, can be found in promoter regions, intergenic regions or other genomic locations that cannot be associated to a specific gene. VarClass is limited to those variants that can be assigned to genes (mainly exonic and intronic variants). Another limitation is that it is not suited to investigate single VUS identified from small-scale experiments, i.e. trio family studies. Genotypes for a large cohort of cases and controls is required for the VarClass models to be constructed. Taking these limitations into consideration, VarClass can provide valuable, complementary information in combination with already available tools that rely on conservation or pathogenicity scores to classify VUS.

VarClass is available as a web interface and provides numerous informative graphical functionalities discussed in Material and Methods. Amongst these, the VarClass web interface is the only tool (to the best of our knowledge) that provides graphical visualization of individualized gene-variant networks for affected and unaffected individuals. This allows for visual comparison between networks from patients with unclear diagnosis and networks from diagnosed patients. Variant assessment as well as risk score prediction for patients of unknown or unclear disease diagnosis (for which GWAS or WES data is available) is also available through the VarClass web interface. We believe that using individualized gene-variant networks and risk prediction for patients of unclear diagnosis through the VarClass web interface may be a valuable tool in performing systemic interpretation of genetic variation data, thus paving the way to individualized diagnosis of patients using genotypic information.

## Material and Methods

### The VarClass analysis pipeline

The VarClass flowchart is shown in Fig. 1. A graphical abstract depiction of Fig. 1, with additional details and guidelines to the user for running the VarClass web server using an example dataset, can be found in Supplementary Information Fig. S5 and the online help documentation.

### Databases and datasets

For the development, validation and performance assessment of the VarClass method a number of well-established databases and available datasets are used.

For the validation of VarClass, two pancreatic cancer datasets are used, comprised of known cancer related variants. Finally, the application of VarClass is assessed using three GWAS datasets and one WES dataset (steps 3–7 from VarClass pipeline). The datasets are downloaded from GEO (https://www.ncbi.nlm.nih.gov/geo/) and the Image & Data Archive (IDA) (https://ida.loni.usc.edu/login.jsp?project = PPMI&page = HOME). The datasets include: (1) Two pancreatic cancer datasets published in Tan A. C. *et. al*.^38^, (2) Two childhood intellectual disability dataset published in Friedman J. M. *et. al*.^53^, (3) One gastric cancer dataset (unpublished study - see GEO) (4) one Parkinson’s whole exome sequencing (WES) dataset obtained by the Parkinson’s Progression Markers Initiative (PPMI). For details, see Supplementary Table S1. Datasets are transformed to a 0, 1, 2 data format whereby each numeric code denotes the number of alleles carried. Therefore, 0-homozygote reference, 1-Heterozygote, 2-Homozygote alternate.

ClinVar was downloaded (date:11/11/2016) and stored locally on our servers as a MySQL database to allow for simple SQL querying. Moreover, the local version of ClinVar is also connected to the VarClass web tool to allow user querying via the web interface.

### Network construction using GeneMANIA

The GeneMANIA (http://genemania.org/) application is used to construct gene-to-gene networks for use in the VarClass pipeline. GeneMANIA defaults are used whereby 20 “related” genes (“Max resultant genes” parameter in GeneMANIA) are used to construct networks (determined based on the input genes’ neighbouring interactions). GeneMANIA is available as a Cytoscape plugin as well as a standalone tool. The latter is downloaded and incorporated into the VarClass analysis pipeline for ease of use and computational speed-up (step 3 in VarClass pipeline). GeneMANIA makes use of publicly available, regularly updated biological datasets to extract relationships between genes and construct gene-to-gene, evidence-based networks. The types of datasets supported by GeneMANIA include: protein-protein interaction (collected primarily from studies found in protein interaction databases, such as BioGRID and PathwayCommons), genetic interactions (collected from original research studies and BioGRID), pathways and reactions (collected from various source databases, such as Reactome and BioCyc, via PathwayCommons), gene and protein expression data (collected from the Gene Expression Omnibus (GEO) and only if associated with a publication). The above mentioned datasets are used to construct five different types of networks:

#### Physical Interaction (Protein-protein interaction network)

Two gene products are linked if they are found to interact in a protein-protein interaction study.

#### Genetic interaction (Genetic interaction network)

Two genes are functionally associated if the effects of perturbing one gene are found to be modified by perturbations to a second gene.

#### Pathways (Common pathways network)

Two gene products are linked if they participate in the same pathway.

#### Co-expression (Gene expression network)

Two genes are linked if their expression levels are similar across conditions in a gene expression study.

#### Co-localization (Localization network)

Two genes are linked if they are both expressed in the same tissue or if their gene products are both identified in the same cellular location.

All networks are loaded in R and analysed using the *igraph*^63^ package for node and edge manipulation, as well as detection of first and second order neighbours.

### Odds Ratio Analysis

The R package PredictABEL^64^ is used to perform odds ratio analysis for obtaining variants with high disease association. The package contains functions used in genetic odds ratio analysis including the univariate odds ratios (OR) of the predictors (*ORunivariate*) function used by VarClass. Variants shown to be more frequent in cases than controls are labelled as *high risk* variants. In contrast to variants being more frequent in controls than cases, which are labelled as *protective* variants.

### Risk score prediction using Linear Logistic Regression Analysis and risk model construction

PredictABEL^64^ is also used to perform linear logistic regression fitting for construction of risk models (using *fitLogRegModel* function), assessing their performance, predicting risks (using *predRisk* function) and obtaining weighted and unweighted risk scores (using *riskScore* function). The package contains functions for the various measures that are commonly used in genetic risk prediction studies to assess model performance, including: the *plotRoc* function for plotting receiver operating characteristic (ROC) curves and calculating area under the curve (AUC) values and the *reclassification* function for reclassification table construction, net reclassification improvement (NRI) and integrated discrimination improvement (IDI) calculations. The NRI and IDI are measures of improvement in model performance and both of them quantify how well a new model reclassifies the data^37^. PredictABEL also includes functions for graphical representation of statistical results such as: risk distributions (*plotRiskDistribution* function), discrimination box plot (*plotDiscriminationBox* function) and predictiveness curves (*plotPredictivenessCurve* function). The glm (generalized linear model) function utilized by PredictABEL is substituted with the bayesglm function available via the *arm* R package^65^.

### Mock Variant Imputation

The R package linkim^39^ was used to simulate “uninformative” (mock) variants. This was achieved by an imputation algorithm which computes the proportions of genotypes (0-Homozygote reference, 1-Heterzygote and 2- Homozygote alternate) in each column (patient sample) of the validation datasets and generates additional (~1600) imputed variants that closely resemble the true patient genotype distributions. These mock variants are assigned to randomly selected genes from the validation datasets, to make sure that the same genes are used for both true variants and the mock variants. This will ensure that the mock variants are placed on the constructed network in a similar manner to the informative (true) variants.

### The VarClass Tool and Web Interface

VarClass is available as a standalone tool implemented for Linux environments (downloadable at http://bioinformatics.cing.ac.cy/downloads/VarClassTool.tar.gz) as well as a user friendly, interactive web interface (http://bioinformatics.cing.ac.cy/varclass/). Due to computational constraints and real time estimations of running the whole VarClass pipeline on large dataset, the web interface only supports a one-by-one analysis of variants from large-scale data. The interface supports all the VarClass functionalities (as well as some extra functionalities) including, sub-network investigation, pathway enrichment, linkage disequilibrium calculations, risk prediction and individualized patient genotype network investigation (see Supplementary Fig. S4). The standalone tool allows for analysis of full datasets and assigns a VarClass score to all variants in the dataset of interest. The user then has the option to upload the list of variants directly into the VarClass web interface for additional online, graphical and interactive capabilities. It should be noted that the VarClass web interface does not rely on the output generated from the VarClass standalone tool; on the contrary, it is fully functional with any list of variants and their associated genes for which genotyping information is available. It is thus also useful for uploading datasets previously analysed using other ranking tools and methodologies and comparing how VarClass scores the variants of these datasets (see online help for details).

## Supplementary information

Supplementary information
