# Supplementary material for: Selecting variants of unknown significance through network-based gene-association significantly improves risk prediction for disease-control cohorts
Source: Sci Rep. 2019 Mar 1;9:3266. doi: 10.1038/s41598-019-39796-w (PMC6397233; doi:10.1038/s41598-019-39796-w)
Supplement: Supplementary file 1 — Supplementary information [file 41598_2019_39796_MOESM1_ESM.docx]

Selecting variants of unknown significance through network-based gene-association significantly improves risk prediction for disease-control cohorts

Anastasis Oulas^1,2*^, George Minadakis^1,2^, Margarita Zachariou^1,2^, George M. Spyrou^1,2^

The Cyprus Institute of Neurology & Genetics, Bioinformatics Group**,**  6 International Airport Avenue, 2370 Nicosia, Cyprus, P.O.Box 23462, 1683 Nicosia, Cyprus.

2 The Cyprus School of Molecular Medicine**,**  6 International Airport Avenue, 2370 Nicosia, Cyprus, P.O.Box 23462, 1683 Nicosia, Cyprus

* Correspondence to anastasioso@cing.ac.cy

**Table S1 – Datasets used for VarClass validation and performance assessment**

| Dataset | Disease Type | Variants | Affected | Control | Type of Data |
| --- | --- | --- | --- | --- | --- |
| GSE8055 | pancreatic cancer | 928 | 141 | 89 | SNP Array |
| GSE8054 | pancreatic cancer | 1189 | 121 | 87 | SNP Array |
| GSE7226 Platform: GPL2005 | intellectual disability | 59015 | 103 | 210 | SNP Array |
| GSE7226 Platform: GPL2004 | intellectual disability | 57299 | 102 | 213 | SNP Array |
| GSE58356 | Gastric cancer | 319283 | 329 | 683 | SNP Array |
| PPMI | Parkinson’s disease | 707051 | 462 | 183 | WES |

| **Table S2. Parkinson’s dataset top scoring variants** | | | | | | | | | | | | |
| --- | --- | --- | --- | --- | --- | --- | --- | --- | --- | --- | --- | --- |
| VariantID | VariantGene | Ave_Pred_Acc | Ave_Sens | Ave_Spec | Ave_AUC_with_Var | Ave_AUC_without_Var | Ave_Var_Zvalue | Ave_Var_Pvalue | Ave_Diff | Ave_IDI | Ave_IDI_pvalue | Network* |
| rs3172404 | CLDN1 | 62.64922 | 71.8677 | 39.30931 | 0.6114 | 0.5566 | 3.2278 | 0.001799 | 0.0548 | 0.03194 | 0.122924 | coexp |
| None | LYRM7 | 59.55205 | 65.39504 | 44.78979 | 0.5642 | 0.5114 | 3.2304 | 3.90259 | 0.0528 | 0.02882 | 0.082494 | coexp |
| rs3745509 | MYH14 | 67.75365 | 85.67789 | 22.44745 | 0.5872 | 0.4814 | 2.7854 | 0.007904 | 0.1058 | 0.02822 | 0.100666 | pi |
| None | P2RY13 | 57.97745 | 64.08135 | 42.58258 | 0.5516 | 0.505 | 3.2118 | 3.88066 | 0.0466 | 0.02632 | 0.170468 | coexp |
| rs594445 | MOCOS | 59.38128 | 67.96634 | 37.67267 | 0.5818 | 0.5388 | 2.9756 | 0.007817 | 0.043 | 0.0259 | 0.245166 | coexp |
| rs2075734 | CBFA2T2 | 62.01219 | 68.62319 | 45.34535 | 0.5954 | 0.5504 | 2.9476 | 0.0061 | 0.045 | 0.02518 | 0.142616 | coexp |
| rs17002187 | CYYR1 | 60.60959 | 67.30949 | 43.70871 | 0.5976 | 0.566 | 3.1726 | 0.004263 | 0.0316 | 0.02518 | 0.172356 | coexp |
| rs73049347 | LYPD4 | 64.65038 | 71.64563 | 47.08709 | 0.6186 | 0.586 | 2.8216 | 0.009834 | 0.0326 | 0.02512 | 0.120402 | coexp |
| rs3745509 | MYH14 | 65.73368 | 75.73866 | 40.48048 | 0.6006 | 0.5654 | 2.9202 | 0.010306 | 0.0352 | 0.02446 | 0.19426 | coexp |
| rs13254035 | FAM83H | 63.24525 | 72.92894 | 38.78378 | 0.5724 | 0.5376 | 3.073 | 0.004535 | 0.0348 | 0.02428 | 0.301542 | coexp |
| rs11547135 | SCARB2 | 63.39803 | 73.35671 | 38.22823 | 0.6052 | 0.5586 | 2.891 | 0.007043 | 0.0466 | 0.02394 | 0.133928 | pi |
| rs1123991 | OR51E2 | 64.34265 | 77.05236 | 32.26727 | 0.5876 | 0.5506 | 2.8842 | 0.005742 | 0.037 | 0.02376 | 0.243204 | coexp |
| rs17500109 | ARL1 | 62.16598 | 70.77373 | 40.39039 | 0.5932 | 0.5588 | 2.7948 | 0.010166 | 0.0344 | 0.0237 | 0.11371 | coexp |
| rs2058205 | KCNA6 | 60.4776 | 67.31183 | 43.25826 | 0.5682 | 0.5308 | 2.8762 | 0.007499 | 0.0374 | 0.02354 | 0.1417 | coexp |
| rs3733156 | ABHD5 | 63.57223 | 73.37073 | 38.75375 | 0.5904 | 0.5536 | 2.825 | 0.022047 | 0.0368 | 0.02346 | 0.187798 | coexp |
| rs4765845 | LRTM2 | 62.47488 | 72.08275 | 38.21321 | 0.5824 | 0.5366 | 2.7586 | 0.019568 | 0.0458 | 0.02248 | 0.167484 | coexp |
| rs3745509 | MYH14 | 64.33186 | 68.82655 | 52.88288 | 0.6264 | 0.5986 | 2.6558 | 0.011683 | 0.0278 | 0.02238 | 0.328238 | pi_coexp |
| rs11481 | CHKA | 63.56783 | 74.45535 | 36.02102 | 0.5874 | 0.545 | 2.5676 | 0.016469 | 0.0424 | 0.02176 | 0.220938 | coexp |
| rs1048628 | SDCBP2 | 65.88702 | 72.95699 | 48.13814 | 0.619 | 0.5874 | 2.798 | 0.010043 | 0.0316 | 0.02168 | 0.185612 | coexp |
| rs138344431 | QRFPR | 62.16957 | 67.31417 | 49.2042 | 0.6322 | 0.6038 | 2.5104 | 0.017469 | 0.0284 | 0.02146 | 0.11438 | coexp |
| rs5554 | SLC8A1 | 56.59164 | 58.43151 | 51.95195 | 0.589 | 0.5406 | 2.7746 | 0.011349 | 0.0484 | 0.02124 | 0.181552 | coloc |
| rs7898331 | PNLIPRP3 | 63.08939 | 72.93361 | 38.1982 | 0.5794 | 0.547 | 2.5578 | 0.017542 | 0.0324 | 0.0212 | 0.208344 | coexp |
| rs11481 | CHKA | 58.60251 | 64.94624 | 42.65766 | 0.5632 | 0.5292 | 2.8772 | 0.008531 | 0.034 | 0.02078 | 0.24653 | coexp_coloc |
| None | QSOX2 | 61.09997 | 68.61384 | 42.05706 | 0.5836 | 0.5502 | 2.534 | 3.9262 | 0.0334 | 0.0202 | 0.245696 | coexp |
| rs4643786 | DCK | 64.18875 | 73.81487 | 39.86486 | 0.5724 | 0.5394 | 2.527 | 0.022308 | 0.033 | 0.02004 | 0.20885 | coexp |
|  |  |  |  |  |  |  |  |  |  |  |  |  |
| **Table S3. Gastric cancer dataset top scoring variants** | | | | | | | | | | | | |
| VariantID | VariantGene | Ave_Pred_Acc | Ave_Sens | Ave_Spec | Ave_AUC_with_Var | Ave_AUC_without_Var | Ave_Var_Zvalue | Ave_Var_Pvalue | Ave_Diff | Ave_IDI | Ave_IDI_pvalue | Network* |
| rs138618143 | CSTF2 | 60.37799 | 65.37529 | 57.98733 | 0.6404 | 0.5218 | 6.538 | 3.47E-09 | 0.1186 | 0.07932 | 0.002286 | coexp |
| rs138618143 | CSTF2 | 62.14856 | 65.65035 | 60.46372 | 0.6484 | 0.5328 | 6.1906 | 8.93E-09 | 0.1156 | 0.07832 | 0.000376 | coexp_coloc_gi |
| rs12008888 | CYLC1 | 61.35834 | 65.97669 | 59.12838 | 0.6304 | 0.5248 | 6.4936 | 9.18E-08 | 0.1056 | 0.07664 | 0.00196 | coexp |
| None | COL4A6 | 61.26372 | 68.68531 | 57.67926 | 0.6532 | 0.5316 | 6.3158 | 1.54E-09 | 0.1216 | 0.07458 | 0.002014 | coloc |
| rs9809030 | DPPA2 | 60.17363 | 67.45921 | 56.65522 | 0.6378 | 0.5238 | 6.284 | 9.89E-10 | 0.114 | 0.0743 | 0.000862 | coexp |
| rs138618143 | CSTF2 | 59.28945 | 62.29837 | 57.83813 | 0.6188 | 0.4878 | 6.153 | 1.37E-08 | 0.131 | 0.074 | 0.030398 | gi |
| rs142786156 | MAGEA12 | 59.88002 | 62.28904 | 58.71619 | 0.621 | 0.492 | 5.8304 | 5.08E-07 | 0.129 | 0.07164 | 0.001324 | coexp |
| rs142786156 | MAGEA12 | 60.77512 | 68.38695 | 57.10498 | 0.6322 | 0.5212 | 6.3016 | 6.33E-08 | 0.111 | 0.07134 | 0.001436 | coloc |
| rs142786156 | MAGEA12 | 59.28216 | 65.65501 | 56.21297 | 0.6298 | 0.5186 | 5.6848 | 1.12E-07 | 0.1112 | 0.06944 | 0.006386 | coexp_coloc |
| rs138618143 | CSTF2 | 61.26334 | 68.07459 | 57.97553 | 0.6328 | 0.4994 | 5.6916 | 3.77E-08 | 0.1334 | 0.0671 | 0.000318 | coloc |
| rs9261468 | TRIM40 | 55.83388 | 58.06527 | 54.75741 | 0.5644 | 0.514 | 3.5448 | 0.00082762 | 0.0504 | 0.02338 | 0.055238 | coexp_gi |
| rs9261468 | TRIM40 | 54.3418 | 58.662 | 52.26707 | 0.5546 | 0.5104 | 3.4844 | 0.00081 | 0.0442 | 0.02176 | 0.26566 | coexp |
|  |  |  |  |  |  |  |  |  |  |  |  |  |
| **Table S4. Intellectual Disability GPL2005 dataset top scoring variants** | | | | | | | | | | | | |
| VariantID | VariantGene | Ave_Pred_Acc | Ave_Sens | Ave_Spec | Ave_AUC_with_Var | Ave_AUC_without_Var | Ave_Var_Zvalue | Ave_Var_Pvalue | Ave_Diff | Ave_IDI | Ave_IDI_pvalue | Network* |
| rs10493766 | DDAH1 | 38.1338 | 35.42857 | 39.43522 | 0.3346 | 0.358 | 0.189 | 0.317732 | -0.0234 | -0.03242 | 0.179048 | coexp_coloc_gi |
| rs9299482 | CTNNA3 | 40.044 | 32.42857 | 43.72093 | 0.36 | 0.3784 | -0.2058 | 0.33024 | -0.0184 | -0.03234 | 0.336814 | coexp |
| rs840699 | HNRPA3 | 39.97312 | 30.28571 | 44.54042 | 0.3242 | 0.3504 | 0.119 | 0.50329 | -0.0262 | -0.02936 | 0.211178 | gi |
| rs4389203 | METTL4 | 37.7421 | 31.2381 | 40.84164 | 0.3434 | 0.3576 | 0.2162 | 0.460774 | -0.0142 | -0.02804 | 0.289194 | gi |
| rs6977649 | TNFAIP9 | 39.40508 | 36.19048 | 40.88594 | 0.3342 | 0.3638 | -0.6532 | 0.532264 | -0.0296 | -0.02798 | 0.294094 | coexp |
| rs3731572 | LTBP1 | 36.50186 | 35.38095 | 37.03212 | 0.3274 | 0.3478 | 0.035 | 0.374306 | -0.0204 | -0.02786 | 0.366306 | coexp_coloc_gi |
| rs10515890 | MAT2B | 45.11905 | 50.85714 | 42.27021 | 0.462 | 0.4724 | 1.029 | 0.37354 | -0.0104 | -0.02782 | 0.291002 | gi |
| rs10512855 | MRPS30 | 41.23784 | 35.47619 | 44.04208 | 0.3546 | 0.3836 | -0.0336 | 0.3745 | -0.029 | -0.02748 | 0.375896 | coexp |
| rs10487661 | TBXAS1 | 40.62852 | 29.33333 | 46.03544 | 0.3156 | 0.343 | 0.6152 | 0.443034 | -0.0274 | -0.02632 | 0.25775 | gi |
| rs9307943 | CTSO | 37.07501 | 38.2381 | 36.567 | 0.3238 | 0.3426 | 0.5282 | 0.200218 | -0.0188 | -0.02604 | 0.41705 | gi |
| rs2150167 | MAMDC1 | 46.34921 | 46.19048 | 46.53378 | 0.4214 | 0.442 | -0.0174 | 0.325745 | -0.0206 | -0.02536 | 0.26685 | gi |
| rs2385624 | EPHA4 | 41.28936 | 46.95238 | 38.5825 | 0.4122 | 0.482 | -0.1014 | 0.38058 | -0.0698 | -0.02522 | 0.248624 | pi |
| rs10503551 | TUSC3 | 40.9615 | 40.33333 | 41.29568 | 0.3702 | 0.4016 | -0.5016 | 0.305788 | -0.0314 | -0.02496 | 0.242054 | coexp |
| rs2013449 | NDN | 33.33333 | 26.38095 | 36.55592 | 0.2874 | 0.3032 | -0.1006 | 0.230978 | -0.0158 | -0.0242 | 0.214474 | gi |
| rs1481187 | RAB28 | 40.91494 | 40.95238 | 40.81949 | 0.3982 | 0.4152 | -0.2862 | 0.335808 | -0.017 | -0.0241 | 0.180502 | coexp |
| rs1648128 | LOC399947 | 39.04554 | 44.14286 | 36.63344 | 0.3916 | 0.4024 | 0.0188 | 0.144762 | -0.0108 | -0.02376 | 0.324952 | coexp |
| rs912826 | PITRM1 | 41.96829 | 40.42857 | 42.75748 | 0.3662 | 0.3728 | -0.0642 | 0.323682 | -0.0066 | -0.02326 | 0.505072 | coexp |
| rs10516488 | BANK1 | 40.63492 | 43.90476 | 38.98117 | 0.3678 | 0.3842 | 0.1226 | 0.247583 | -0.0164 | -0.02306 | 0.433506 | coexp_gi |
| rs998133 | PRKG1 | 39.94816 | 35.28571 | 42.22591 | 0.3616 | 0.3806 | 0.0918 | 0.26442 | -0.019 | -0.02302 | 0.258096 | coexp |
| rs816278 | C10orf117 | 41.55626 | 40 | 42.24806 | 0.3732 | 0.383 | 0.254 | 0.127556 | -0.0098 | -0.02294 | 0.544946 | gi |
| rs10498936 | C6orf157 | 40.34082 | 43.14286 | 39.0144 | 0.3662 | 0.389 | 0.1598 | 0.455744 | -0.0228 | -0.02282 | 0.465504 | gi |
| rs10498010 | SPAG16 | 40.00384 | 39.33333 | 40.32115 | 0.3554 | 0.3708 | 0.0894 | 0.50767 | -0.0154 | -0.02246 | 0.264376 | gi |
| rs10510227 | CNTN4 | 41.2476 | 40.19048 | 41.74972 | 0.3582 | 0.3796 | 0.0434 | 0.16077 | -0.0214 | -0.02242 | 0.320882 | coexp |
| rs2008521 | CARD8 | 39.63582 | 34.19048 | 42.15947 | 0.3338 | 0.35 | 0.47 | 0.34067 | -0.0162 | -0.02236 | 0.279806 | coexp |
| rs2452199 | IDI2 | 40.2509 | 39.04762 | 40.77519 | 0.3436 | 0.3622 | -1.0144 | 0.332849 | -0.0186 | -0.02232 | 0.248404 | coexp_coloc_gi |
| rs280302 | TFAP2D | 37.44384 | 35.19048 | 38.51606 | 0.3276 | 0.3406 | -0.562 | 0.449387 | -0.013 | -0.02222 | 0.258232 | coexp_gi |
| rs230489 | SLC39A8 | 41.59146 | 42.09524 | 41.3289 | 0.4034 | 0.4204 | -0.5256 | 0.30328 | -0.017 | -0.02216 | 0.251162 | coexp_gi |
| rs4667272 | FLJ12519 | 40.34706 | 40.90476 | 39.94463 | 0.3296 | 0.3466 | -0.7942 | 0.286124 | -0.017 | -0.02184 | 0.264076 | gi |
| rs10496448 | IL1F10 | 41.6017 | 34.42857 | 45.09413 | 0.4032 | 0.4152 | -0.2192 | 0.285368 | -0.012 | -0.02182 | 0.442488 | coexp |
| rs7099088 | VTI1A | 40.03296 | 37.28571 | 41.33998 | 0.3516 | 0.3698 | -0.3532 | 0.218938 | -0.0182 | -0.02168 | 0.277908 | coexp_gi |
| rs1374802 | BTD | 38.75304 | 46.14286 | 35.20487 | 0.3592 | 0.379 | 0.0788 | 0.31956 | -0.0198 | -0.02154 | 0.36322 | gi |
| rs10517035 | ATP8A1 | 39.32924 | 39.19048 | 39.41307 | 0.3952 | 0.418 | 0.3478 | 0.384688 | -0.0228 | -0.02152 | 0.284534 | coexp |
| rs1332776 | FLJ12439 | 35.85701 | 34.38095 | 36.62237 | 0.3474 | 0.3638 | 0.4538 | 0.370578 | -0.0164 | -0.0215 | 0.499456 | coexp |
| rs6765602 | SLC9A9 | 39.71102 | 33.47619 | 42.72425 | 0.356 | 0.3756 | -0.506 | 0.387244 | -0.0196 | -0.02128 | 0.280244 | gi |
| rs1562111 | ENC1 | 39.06698 | 42.19048 | 37.58583 | 0.3454 | 0.3612 | -0.187 | 0.28784 | -0.0158 | -0.02126 | 0.227354 | coexp |
| rs483089 | RPS28 | 40.34178 | 36.38095 | 42.25914 | 0.3794 | 0.3954 | -1.0334 | 0.445089 | -0.016 | -0.02124 | 0.481894 | gi |
| rs10497268 | TTC21B | 37.49952 | 39.2381 | 36.67774 | 0.3264 | 0.3442 | 0.04 | 0.25718 | -0.0178 | -0.0212 | 0.309442 | gi |
| rs807894 | QKI | 40.29122 | 42.09524 | 39.36877 | 0.3698 | 0.3882 | 0.7474 | 0.468463 | -0.0184 | -0.02112 | 0.465498 | pi_coexp_gi |
| rs871476 | ANAPC4 | 35.2381 | 34.38095 | 35.70321 | 0.301 | 0.3144 | 1.0314 | 0.361864 | -0.0134 | -0.02096 | 0.4744 | pi_coexp_gi |
| rs4833432 | CAMK2D | 38.71432 | 33.38095 | 41.30676 | 0.3226 | 0.334 | -1.285 | 0.390096 | -0.0114 | -0.02084 | 0.398448 | gi |
| rs9287097 | PACE-1 | 41.8686 | 32.33333 | 46.41196 | 0.3656 | 0.3756 | -0.054 | 0.390816 | -0.01 | -0.02062 | 0.394996 | coexp |
| rs35639 | CCT2 | 40.65924 | 34.28571 | 43.66556 | 0.3526 | 0.3678 | 0.1002 | 0.443798 | -0.0152 | -0.02054 | 0.220264 | gi |
| rs1860761 | FLJ20701 | 42.24174 | 39.38095 | 43.65449 | 0.3864 | 0.4036 | 1.026 | 0.335414 | -0.0172 | -0.02046 | 0.332366 | coexp_gi |
| rs7176364 | FBN1 | 38.09892 | 31.52381 | 41.33998 | 0.3532 | 0.3672 | -0.1428 | 0.243952 | -0.014 | -0.02044 | 0.543072 | coexp |
| rs2301035 | CBLB | 40.60244 | 41.04762 | 40.36545 | 0.345 | 0.3652 | -0.3202 | 0.532344 | -0.0202 | -0.02036 | 0.409916 | coexp_gi |
| rs2451110 | TSPYL5 | 41.29368 | 38.2381 | 42.75748 | 0.3644 | 0.3816 | 0.3252 | 0.410334 | -0.0172 | -0.02036 | 0.370022 | gi |
| rs10520196 | AADAT | 36.80876 | 36.19048 | 37.09856 | 0.3088 | 0.3116 | -0.8228 | 0.364463 | -0.0028 | -0.02032 | 0.41151 | coexp_coloc_gi |
| rs9321987 | UTRN | 38.07396 | 38.04762 | 37.97342 | 0.38 | 0.3932 | 0.054 | 0.420638 | -0.0132 | -0.02032 | 0.189282 | pi_coexp_path |
| rs40321 | NEDD1 | 41.24776 | 44.04762 | 39.87818 | 0.3542 | 0.3638 | -0.765 | 0.33002 | -0.0096 | -0.0203 | 0.46149 | coexp_path_gi |
| rs7888371 | FGF13 | 43.13892 | 36.14286 | 46.43411 | 0.377 | 0.3946 | -0.335 | 0.40824 | -0.0176 | -0.0202 | 0.386508 | coexp_path_gi |
| rs4493980 | TLE4 | 39.98416 | 37.19048 | 41.29568 | 0.3568 | 0.3688 | -0.0032 | 0.285728 | -0.012 | -0.02008 | 0.473184 | coexp_path_gi |
| **Table S5. Intellectual Disability GPL2004 dataset top scoring variants** | | | | | | | | | | | | |
| VariantID | VariantGene | Ave_Pred_Acc | Ave_Sens | Ave_Spec | Ave_AUC_with_Var | Ave_AUC_without_Var | Ave_Var_Zvalue | Ave_Var_Pvalue | Ave_Diff | Ave_IDI | Ave_IDI_pvalue | Network* |
| rs10494979 | PTPN14 | 38.33077 | 33.95238 | 40.47619 | 0.369 | 0.3988 | 0.4962 | 0.247788 | -0.0298 | -0.03682 | 0.364186 | coexp |
| rs3916092 | EFHB | 48.55095 | 47.61905 | 49.04762 | 0.4136 | 0.4404 | 0.6246 | 0.306602 | -0.0268 | -0.03672 | 0.351558 | coexp_gi |
| rs10517234 | PCDH7 | 43.0978 | 37.66667 | 45.71429 | 0.383 | 0.4094 | -0.4644 | 0.65297 | -0.0264 | -0.03258 | 0.489458 | coexp_path_gi |
| rs6974138 | POM121 | 41.17256 | 43.42857 | 40 | 0.4068 | 0.4296 | 0.329 | 0.533064 | -0.0228 | -0.03058 | 0.337016 | coexp_path_gi |
| rs4825029 | MAGEC3 | 44.73118 | 44.71429 | 44.7619 | 0.4196 | 0.4498 | -0.6414 | 0.216654 | -0.0302 | -0.0302 | 0.251742 | coexp |
| rs10495872 | FLJ32954 | 38.66871 | 32.95238 | 41.42857 | 0.3022 | 0.3228 | 1.3032 | 0.215333 | -0.0206 | -0.02984 | 0.2233 | coexp |
| rs10507661 | PCDH20 | 41.84844 | 39.7619 | 42.85714 | 0.4098 | 0.4278 | 0.049 | 0.895788 | -0.018 | -0.02782 | 0.407644 | coexp |
| rs10503367 | AGPAT5 | 43.76856 | 46.42857 | 42.38095 | 0.4146 | 0.4346 | -0.6282 | 0.382742 | -0.02 | -0.0277 | 0.37357 | gi |
| rs10513904 | ZNF519 | 43.78392 | 39.90476 | 45.71429 | 0.3918 | 0.4124 | -0.3128 | 0.376199 | -0.0206 | -0.0273 | 0.20409 | coexp |
| rs1375255 | KHDRBS3 | 43.80952 | 44.85714 | 43.33333 | 0.4116 | 0.4292 | -0.6614 | 0.198156 | -0.0176 | -0.0271 | 0.459714 | coexp_coloc_gi |
| rs2442359 | FREM2 | 42.46288 | 39.7619 | 43.80952 | 0.3702 | 0.388 | -0.8938 | 0.453624 | -0.0178 | -0.02678 | 0.382316 | coexp |
| rs6610546 | DDX3X | 35.14593 | 29.09524 | 38.09524 | 0.3022 | 0.321 | -0.3462 | 0.271844 | -0.0188 | -0.02658 | 0.218092 | coexp |
| rs10511522 | TYRP1 | 38.99642 | 43.66667 | 36.66667 | 0.3746 | 0.392 | 0.5834 | 0.134508 | -0.0174 | -0.02646 | 0.381224 | gi |
| rs10509283 | CTNNA3 | 41.81772 | 43.42857 | 40.95238 | 0.3886 | 0.4082 | -0.6262 | 0.612905 | -0.0196 | -0.02624 | 0.303564 | coexp |
| rs9286849 | RGL1 | 41.8638 | 46.61905 | 39.52381 | 0.3924 | 0.4038 | 0.7204 | 0.258808 | -0.0114 | -0.026 | 0.237284 | gi |
| rs229365 | NCAM2 | 40.26626 | 40.7619 | 40 | 0.3634 | 0.3812 | 0.608 | 0.266938 | -0.0178 | -0.0256 | 0.40683 | coexp_coloc_gi |
| rs1113480 | PTPRJ | 47.30159 | 46.61905 | 47.61905 | 0.449 | 0.461 | -1.034 | 0.297544 | -0.012 | -0.02558 | 0.262902 | gi |
| rs10514969 | HTR1A | 41.84332 | 41.61905 | 41.90476 | 0.3664 | 0.3856 | 0.3162 | 0.299368 | -0.0192 | -0.02538 | 0.699494 | coexp |
| rs10511642 | ADAMTSL1 | 42.82642 | 42.7619 | 42.85714 | 0.4082 | 0.4266 | 0.884 | 0.329617 | -0.0184 | -0.02536 | 0.292686 | coexp |
| rs10493776 | COL24A1 | 37.36815 | 38.71429 | 36.66667 | 0.347 | 0.3612 | 0.7162 | 0.271034 | -0.0142 | -0.02518 | 0.321282 | gi |
| rs1535891 | EPSTI1 | 45.71429 | 42.85714 | 47.14286 | 0.3956 | 0.41 | -0.1206 | 0.453763 | -0.0144 | -0.02508 | 0.461532 | coexp |
| rs10503079 | BCL2 | 43.16436 | 42.61905 | 43.33333 | 0.398 | 0.412 | -0.4854 | 0.31092 | -0.014 | -0.02496 | 0.209994 | coexp_gi |
| rs10510582 | LRRC3B | 41.85356 | 33.19048 | 46.19048 | 0.3284 | 0.3424 | 0.0976 | 0.402041 | -0.014 | -0.024 | 0.204778 | gi |
| rs10517639 | PDGFC | 42.81618 | 43.85714 | 42.38095 | 0.4082 | 0.4238 | 1.1722 | 0.479526 | -0.0156 | -0.02372 | 0.290408 | coexp |
| rs6574492 | NRXN1 | 44.74654 | 45.57143 | 44.28571 | 0.4236 | 0.453 | -0.9696 | 0.450289 | -0.0294 | -0.02318 | 0.23381 | pi |
| rs997641 | COL24A1 | 39.61086 | 39.95238 | 39.52381 | 0.3834 | 0.4004 | 0.0422 | 0.329121 | -0.017 | -0.02318 | 0.266632 | coexp_gi |
| rs10515304 | SLCO4C1 | 43.4511 | 41.71429 | 44.28571 | 0.433 | 0.4448 | 0.1554 | 0.3804 | -0.0118 | -0.02298 | 0.14767 | coexp_gi |
| rs4891384 | RTTN | 40.29698 | 32.38095 | 44.28571 | 0.3744 | 0.3886 | -0.5278 | 0.274692 | -0.0142 | -0.02296 | 0.34576 | gi |
| rs3010054 | RGL1 | 40.2765 | 34.90476 | 42.85714 | 0.3492 | 0.3628 | 0.163 | 0.297192 | -0.0136 | -0.0229 | 0.437054 | coexp |
| rs10509872 | SORCS1 | 43.45622 | 44.52381 | 42.85714 | 0.4374 | 0.4596 | -0.3164 | 0.227573 | -0.0222 | -0.02272 | 0.30065 | coexp_gi |
| rs10486449 | C7orf9 | 46.95853 | 50.61905 | 45.2381 | 0.4362 | 0.4554 | -0.561 | 0.24311 | -0.0192 | -0.0227 | 0.510776 | coexp_gi |
| rs1379641 | C18orf34 | 42.10445 | 40.42857 | 42.85714 | 0.395 | 0.4028 | -1.3648 | 0.2814 | -0.0078 | -0.02258 | 0.573762 | coexp |
| rs1542689 | GUCY1A2 | 45.6938 | 44.42857 | 46.19048 | 0.4306 | 0.4632 | -0.4542 | 0.398768 | -0.0326 | -0.0224 | 0.36159 | pi |
| rs10491900 | GLIS3 | 38.37174 | 33.19048 | 40.95238 | 0.3512 | 0.3686 | -0.419 | 0.408839 | -0.0174 | -0.02238 | 0.133066 | coexp_gi |
| rs1982756 | TREML4 | 41.21864 | 42.7619 | 40.47619 | 0.3616 | 0.3714 | 0.7856 | 0.505752 | -0.0098 | -0.02228 | 0.197114 | coexp_gi |
| rs10504207 | PENK | 38.04916 | 34.2381 | 40 | 0.3394 | 0.3496 | -0.4048 | 0.439854 | -0.0102 | -0.0222 | 0.393856 | gi |
| rs544080 | UGCGL2 | 42.82642 | 43.71429 | 42.38095 | 0.4118 | 0.425 | -1.1362 | 0.253294 | -0.0132 | -0.0222 | 0.260378 | gi |
| rs1926559 | TECTB | 40.58372 | 33.09524 | 44.28571 | 0.3468 | 0.3594 | -0.2384 | 0.258235 | -0.0126 | -0.02218 | 0.198784 | coexp_gi |
| rs10508031 | MBNL2 | 40.8807 | 41.71429 | 40.47619 | 0.3924 | 0.4118 | -0.33 | 0.355708 | -0.0194 | -0.02216 | 0.18312 | coexp |
| rs10505365 | ENPP2 | 36.75883 | 36.7619 | 36.66667 | 0.359 | 0.3716 | 0.4638 | 0.396319 | -0.0126 | -0.02212 | 0.565918 | coexp_coloc_gi |
| rs10509887 | SORCS1 | 43.76344 | 46.52381 | 42.38095 | 0.4006 | 0.4124 | -0.483 | 0.464554 | -0.0118 | -0.0221 | 0.401444 | coexp |
| rs1414141 | TNC | 41.19816 | 42.71429 | 40.47619 | 0.3788 | 0.3984 | -0.4374 | 0.2999 | -0.0196 | -0.02202 | 0.369962 | gi |
| rs9283612 | IL12A | 43.44598 | 51.42857 | 39.52381 | 0.4092 | 0.4302 | -0.063 | 0.339392 | -0.021 | -0.02196 | 0.245772 | gi |
| rs3123045 | PLA2G4A | 39.59549 | 40.71429 | 39.04762 | 0.3672 | 0.3754 | 0.1676 | 0.84347 | -0.0082 | -0.0219 | 0.476692 | coexp_gi |
| rs10483025 | C21orf18 | 46.66667 | 48.66667 | 45.71429 | 0.4336 | 0.4484 | -0.0636 | 0.258367 | -0.0148 | -0.0218 | 0.295644 | gi |
| rs2177065 | RYR2 | 42.83154 | 42.57143 | 42.85714 | 0.3702 | 0.3792 | 0.6626 | 0.561212 | -0.009 | -0.0218 | 0.403072 | coexp_coloc_path_gi |
| rs2405755 | SI | 40.93702 | 37.19048 | 42.85714 | 0.3654 | 0.3812 | -0.1844 | 0.213322 | -0.0158 | -0.02174 | 0.369708 | coexp_gi |
| rs337743 | CBLN2 | 39.26779 | 38.80952 | 39.52381 | 0.3542 | 0.369 | -0.2832 | 0.334636 | -0.0148 | -0.0216 | 0.613332 | coexp |
| rs4078278 | PARD3 | 42.78546 | 47.57143 | 40.47619 | 0.4192 | 0.4298 | -0.792 | 0.467182 | -0.0106 | -0.02156 | 0.439856 | gi |
| rs4128358 | VGCNL1 | 35.79109 | 32.09524 | 37.61905 | 0.3226 | 0.3364 | -0.0908 | 0.380462 | -0.0138 | -0.02154 | 0.267506 | coexp_gi |
| rs10506533 | WIF1 | 43.149 | 49.66667 | 40 | 0.4122 | 0.4212 | 0.3384 | 0.426287 | -0.009 | -0.02144 | 0.446718 | coexp_coloc_gi |
| rs10485685 | PTPRT | 39.93856 | 37.66667 | 40.95238 | 0.3592 | 0.3776 | 0.7494 | 0.25039 | -0.0184 | -0.02138 | 0.23855 | coexp |
| rs1491491 | NRXN3 | 42.18126 | 37.90476 | 44.28571 | 0.3842 | 0.3964 | -0.3672 | 0.42422 | -0.0122 | -0.02136 | 0.263682 | coexp |
| rs10512749 | ADAMTS16 | 37.08141 | 38.85714 | 36.19048 | 0.328 | 0.343 | -0.319 | 0.432295 | -0.015 | -0.0213 | 0.387344 | gi |
| rs2311139 | XYLT1 | 40.23041 | 35.7619 | 42.38095 | 0.3508 | 0.3656 | -0.475 | 0.366896 | -0.0148 | -0.0213 | 0.38365 | coexp |
| rs893554 | COX7C | 39.59037 | 40.52381 | 39.04762 | 0.3594 | 0.3726 | -1.0552 | 0.421302 | -0.0132 | -0.02124 | 0.10886 | coexp |
| rs10502066 | AASDHPPT | 41.22376 | 49.57143 | 37.14286 | 0.3628 | 0.3786 | -0.7032 | 0.262848 | -0.0158 | -0.02114 | 0.545326 | coexp |
| rs1388180 | NS3TP2 | 43.44086 | 44.61905 | 42.85714 | 0.3814 | 0.4012 | 0.8658 | 0.116541 | -0.0198 | -0.02114 | 0.531578 | gi |
| rs721471 | DDAH1 | 38.31541 | 38.7619 | 38.09524 | 0.3526 | 0.3628 | 0.4222 | 0.517854 | -0.0102 | -0.0211 | 0.61438 | gi |
| rs4737588 | ASPH | 44.70046 | 44.2381 | 44.7619 | 0.4152 | 0.434 | -0.1622 | 0.442746 | -0.0188 | -0.0209 | 0.471518 | gi |
| rs10513830 | LPP | 43.11828 | 49.42857 | 40 | 0.424 | 0.4394 | 0.1618 | 0.367294 | -0.0154 | -0.02088 | 0.221838 | pi_coexp_gi |
| rs9301083 | DAOA | 39.32924 | 36.2381 | 40.95238 | 0.364 | 0.3786 | -0.3724 | 0.360252 | -0.0146 | -0.0208 | 0.399852 | coexp |
| rs10521686 | PGRMC1 | 38.66359 | 39.85714 | 38.09524 | 0.3698 | 0.3852 | -0.7568 | 0.370876 | -0.0154 | -0.02078 | 0.288536 | gi |
| rs10488764 | FDX1 | 38.30005 | 34.71429 | 40 | 0.3374 | 0.3468 | 0.1518 | 0.447678 | -0.0094 | -0.02076 | 0.361494 | gi |
| rs10486911 | GNAI1 | 44.383 | 42.52381 | 45.2381 | 0.3858 | 0.398 | 0.4194 | 0.528684 | -0.0122 | -0.0207 | 0.34501 | gi |
| rs1886039 | LCP1 | 39.60061 | 36.80952 | 40.95238 | 0.359 | 0.3696 | -0.3076 | 0.342132 | -0.0106 | -0.02066 | 0.439334 | gi |
| rs1407127 | RABGAP1L | 42.49872 | 47.42857 | 40 | 0.4054 | 0.4198 | 0.3854 | 0.384836 | -0.0144 | -0.02064 | 0.431118 | gi |
| rs4143127 | CNTN4 | 39.95904 | 43.90476 | 38.09524 | 0.3858 | 0.3946 | -0.1914 | 0.32415 | -0.0088 | -0.0206 | 0.42967 | coexp_gi |
| rs10512755 | ADAMTS16 | 44.09626 | 47.57143 | 42.38095 | 0.4288 | 0.4386 | 0.6952 | 0.521486 | -0.0098 | -0.02046 | 0.330766 | coexp |
| rs1371231 | VEGFC | 43.44598 | 44.61905 | 42.85714 | 0.432 | 0.4386 | -0.4286 | 0.301153 | -0.0066 | -0.02038 | 0.247872 | gi |
| rs636074 | RAB31 | 41.87404 | 44.66667 | 40.47619 | 0.3804 | 0.3962 | -0.521 | 0.446668 | -0.0158 | -0.02038 | 0.499042 | gi |
| rs3864588 | LPHN2 | 38.96057 | 37.80952 | 39.52381 | 0.3448 | 0.359 | 0.3992 | 0.36194 | -0.0142 | -0.02034 | 0.407346 | coexp |
| rs2504080 | MOCS1 | 42.1659 | 41.71429 | 42.38095 | 0.3914 | 0.4086 | -0.0654 | 0.38797 | -0.0172 | -0.0203 | 0.457792 | gi |
| rs1858466 | CA10 | 47.89555 | 50.47619 | 46.66667 | 0.439 | 0.464 | -0.5174 | 0.415152 | -0.025 | -0.02024 | 0.188914 | coexp |
| rs10493588 | ASB17 | 45.064 | 46.52381 | 44.28571 | 0.397 | 0.4186 | 0.4676 | 0.333008 | -0.0216 | -0.02022 | 0.28152 | gi |
| rs951933 | RPS28 | 39.30364 | 35.09524 | 41.42857 | 0.3586 | 0.3704 | 0.3686 | 0.417893 | -0.0118 | -0.0202 | 0.21541 | coexp |
| rs7225041 | FLJ10979 | 42.51408 | 40.80952 | 43.33333 | 0.373 | 0.383 | 0.1566 | 0.258058 | -0.01 | -0.02018 | 0.422812 | coexp |
| rs10502933 | RKHD2 | 39.61598 | 42.47619 | 38.09524 | 0.385 | 0.3944 | -0.2462 | 0.483115 | -0.0094 | -0.02016 | 0.381536 | gi |
| rs708629 | C20orf20 | 42.15054 | 41.7619 | 42.38095 | 0.3872 | 0.4026 | 0.3002 | 0.33126 | -0.0154 | -0.0201 | 0.282898 | coexp_gi |
| rs1031505 | ARGBP2 | 42.4424 | 41.66667 | 42.85714 | 0.3746 | 0.3804 | 1.1926 | 0.275082 | -0.0058 | -0.02008 | 0.362526 | coexp_coloc_gi |
| rs10499933 | SHFM1 | 39.30364 | 32.14286 | 42.85714 | 0.3316 | 0.3514 | -0.0656 | 0.337126 | -0.0198 | -0.02008 | 0.223642 | gi |
| rs1387381 | CDH13 | 44.09626 | 34 | 49.04762 | 0.3762 | 0.3978 | 0 | 1 | -0.0216 | -0.02002 | 0.362634 | path |

*VariantID=Variant dbSNP ID (where available), VariantGene=Gene associated with variant, Ave_Pred_Acc=Average prediction accuracy (over 5-fold cross validation), Ave_Sens=Average Sensitivity (over 5-fold cross validation), Ave_Spec=Average Specificity (over 5-fold cross validation), Ave_AUC_with_Var=Average AUC generated from Model 1 runs (over 5-fold cross validation), Ave_AUC_without_Var= Average AUC generated from Model 2 runs (over 5-fold cross validation), Ave_Var_Zvalue= Average variant Z-value from linear regression models (over 5-fold cross validation), Ave_Var_Pvalue= Average variant coefficient p-value from linear regression models (over 5-fold cross validation), Ave_Diff=Difference between Ave_Sens and Ave_Spec, Ave_IDI= Average IDI (over 5-fold cross validation), Ave_IDI_pvalue= Average IDI (over 5-fold cross validation), Networks used as backbone for gene variant analysis: protein-protein interactions (ppi), co-expression (coexp), genetic interaction (gi), co-localization (coloc) and common pathways (cpath).

| rsID | Estimate | Std. Error | z value | Pr(>\|z\|) | Sig. |
| --- | --- | --- | --- | --- | --- |
| rs1373419 | 0.36825 | 0.33014 | 1.115 | 0.26467 |  |
| rs11554507 | -0.62696 | 0.35072 | -1.788 | 0.073833 | . |
| rs3172404 | 0.80401 | 0.2394 | 3.358 | 0.000784 | *** |
| rs201360644 | 0.1459 | 1.38722 | 0.105 | 0.916238 |  |
| rs2245804 | 0.06638 | 1.27446 | 0.052 | 0.958463 |  |
| rs11326 | -0.27013 | 0.26782 | -1.009 | 0.313145 |  |
| rs71653629 | 0.14481 | 0.26217 | 0.552 | 0.580704 |  |
| rs57534886 | 0.37397 | 0.30911 | 1.21 | 0.226344 |  |
| rs12541126 | 0.54728 | 0.25747 | 2.126 | 0.033536 | * |
| rs17310286 | 0.16752 | 1.34521 | 0.125 | 0.900897 |  |
| rs1042992 | 0.16752 | 1.34521 | 0.125 | 0.900897 |  |
| None | 0.10089 | 1.69927 | 0.059 | 0.952654 |  |
| rs9621461 | 0.18439 | 0.27798 | 0.663 | 0.507122 |  |

**Table S6. Parkinson’s rs3172404 synergistic partners and scores**

**Table S7. Gastric Cancer rs138618143 synergistic partners and scores**

| rsID | Estimate | Std. Error | z value | Pr(>\|z\|) | Sig. |
| --- | --- | --- | --- | --- | --- |
| rs3734116 | 0.29036 | 0.381407 | 0.761 | 0.44649 |  |
| rs9640663 | 0.174456 | 0.235461 | 0.741 | 0.45875 |  |
| rs189424344 | -0.17177 | 1.177801 | -0.146 | 0.88405 |  |
| rs11016076 | -0.06683 | 0.180227 | -0.371 | 0.71079 |  |
| rs1800956 | 0.181519 | 0.25973 | 0.699 | 0.48463 |  |
| rs61739617 | 1.756016 | 1.699837 | 1.033 | 0.30158 |  |
| rs12218638 | -0.40523 | 0.249726 | -1.623 | 0.10465 |  |
| rs16956461 | 0.655143 | 0.416921 | 1.571 | 0.11609 |  |
| rs149458030 | 2.027938 | 1.592856 | 1.273 | 0.20297 |  |
| rs141776380 | 0.84526 | 0.508425 | 1.663 | 0.09641 | . |
| rs35686369 | -0.51128 | 0.238918 | -2.14 | 0.03236 | * |
| rs2303080 | 0.595627 | 0.230518 | 2.584 | 0.00977 | ** |
| rs77542170 | 0.962139 | 0.570883 | 1.685 | 0.09192 | . |
| rs199848449 | 0.388862 | 0.640428 | 0.607 | 0.54372 |  |
| rs117795868 | 0.85786 | 1.045409 | 0.821 | 0.41188 |  |
| None | 0.005866 | 0.181235 | 0.032 | 0.97418 |  |
| rs142253103 | 0.149133 | 0.784547 | 0.19 | 0.84924 |  |
| rs146242251 | -0.11924 | 0.554702 | -0.215 | 0.82979 |  |
| rs201126407 | 0.6604 | 0.991797 | 0.666 | 0.5055 |  |
| rs138618143 | 0.676679 | 0.098738 | 6.853 | 7.22E-12 | *** |
| rs11601310 | -0.17272 | 0.18317 | -0.943 | 0.34571 |  |
| rs1532268 | 0.233787 | 0.201184 | 1.162 | 0.24521 |  |
| rs2387326 | -0.03417 | 0.200225 | -0.171 | 0.86451 |  |
| rs34750407 | 0.630674 | 0.26025 | 2.423 | 0.01538 | * |
| rs7834383 | 0.029863 | 0.353603 | 0.084 | 0.93269 |  |
| rs9929218 | 0.337767 | 0.193195 | 1.748 | 0.08041 | . |
| rs2460362 | 0.308806 | 0.222712 | 1.387 | 0.16557 |  |
| rs62333891 | 0.061328 | 0.263894 | 0.232 | 0.81623 |  |

**Table S8. Intellectual Disability GPL2005 rs10494979 synergistic partners and scores**

| rsID | Estimate | Std. Error | z value | Pr(>\|z\|) | Sig. |
| --- | --- | --- | --- | --- | --- |
| rs841396 | -8.52E-01 | 7.22E-01 | -1.18 | 0.23803 |  |
| rs10494979 | 1.24E+00 | 4.74E-01 | 2.623 | 0.00871 | ** |
| rs10494978 | -2.59E-01 | 6.82E-01 | -0.379 | 0.70443 |  |
| rs10495453 | 1.94E+00 | 6.90E-01 | 2.808 | 0.00498 | ** |
| rs7478221 | 1.38E-01 | 4.41E-01 | 0.312 | 0.75503 |  |
| rs2251038 | -8.35E-01 | 1.57E+00 | -0.532 | 0.59454 |  |
| rs1896404 | -3.95E-01 | 3.71E-01 | -1.065 | 0.28699 |  |
| rs10488696 | -2.42E-01 | 5.59E-01 | -0.433 | 0.66478 |  |
| rs1939991 | -3.50E-01 | 4.23E-01 | -0.828 | 0.40794 |  |
| rs10492136 | -4.62E-01 | 6.17E-01 | -0.749 | 0.45377 |  |
| rs2284405 | -4.63E-01 | 8.77E-01 | -0.529 | 0.5971 |  |
| rs10492138 | 6.51E-01 | 4.47E-01 | 1.458 | 0.14474 |  |
| rs2364871 | -2.08E-01 | 7.46E-01 | -0.279 | 0.78032 |  |
| rs10492430 | -3.16E-01 | 1.56E+00 | -0.203 | 0.8395 |  |
| rs10492431 | 3.16E-01 | 1.56E+00 | 0.203 | 0.8395 |  |
| rs874150 | 5.12E-01 | 1.43E+00 | 0.359 | 0.7196 |  |
| rs9319258 | 0.00E+00 | 2.50E+00 | 0 | 1 |  |
| rs10492846 | -3.14E-01 | 6.93E-01 | -0.453 | 0.65075 |  |
| rs9302409 | 2.09E+00 | 8.53E-01 | 2.449 | 0.01431 | * |
| rs10500367 | -6.61E-02 | 4.46E-01 | -0.148 | 0.88215 |  |
| rs10521327 | -7.37E-01 | 7.75E-01 | -0.951 | 0.34174 |  |
| rs7187550 | 1.20E-01 | 5.09E-01 | 0.236 | 0.81371 |  |
| rs4129486 | -6.78E-01 | 1.02E+00 | -0.664 | 0.50649 |  |
| rs1144613 | 2.96E-01 | 3.83E-01 | 0.772 | 0.4399 |  |
| rs7242438 | -7.03E-01 | 3.85E-01 | -1.824 | 0.06816 | . |
| rs10502864 | 2.87E-01 | 5.00E-01 | 0.574 | 0.56605 |  |
| rs10520481 | -7.17E-01 | 7.52E-01 | -0.954 | 0.34019 |  |
| rs6756311 | 8.11E-02 | 3.91E-01 | 0.208 | 0.83553 |  |
| rs5754499 | -2.13E-01 | 1.74E+00 | -0.123 | 0.9025 |  |
| rs10483184 | 1.10E-01 | 7.33E-01 | 0.15 | 0.88067 |  |
| rs10483176 | 3.20E-15 | 2.50E+00 | 0 | 1 |  |
| rs5754496 | 2.13E-01 | 1.74E+00 | 0.123 | 0.9025 |  |
| rs9306283 | 2.26E-15 | 2.50E+00 | 0 | 1 |  |
| rs2172948 | -2.87E-01 | 1.54E+00 | -0.186 | 0.85233 |  |
| rs10510710 | 2.87E-01 | 1.54E+00 | 0.186 | 0.85233 |  |
| rs10510712 | -1.07E-02 | 8.60E-01 | -0.012 | 0.99012 |  |
| rs10510713 | 2.20E-15 | 2.50E+00 | 0 | 1 |  |
| rs10490822 | 1.56E+00 | 1.12E+00 | 1.388 | 0.16528 |  |
| rs830604 | -1.65E-01 | 3.71E-01 | -0.444 | 0.65709 |  |
| rs10514727 | -5.20E-02 | 6.93E-01 | -0.075 | 0.94014 |  |
| rs9310195 | -7.73E-01 | 5.56E-01 | -1.39 | 0.16458 |  |
| rs953304 | -1.65E+00 | 1.18E+00 | -1.399 | 0.16184 |  |
| rs10514307 | 0.00E+00 | 2.50E+00 | 0 | 1 |  |
| rs9293513 | 1.78E+00 | 2.23E+00 | 0.801 | 0.42294 |  |
| rs7760434 | -1.14E+00 | 9.45E-01 | -1.203 | 0.22906 |  |
| rs1856133 | -8.34E-01 | 4.06E-01 | -2.057 | 0.03964 | * |
| rs1340282 | 1.21E-01 | 7.06E-01 | 0.172 | 0.86359 |  |
| rs4317419 | 4.80E-01 | 1.97E+00 | 0.244 | 0.80736 |  |
| rs967992 | 2.39E-15 | 2.50E+00 | 0 | 1 |  |
| rs724845 | -5.37E-01 | 5.32E-01 | -1.01 | 0.31246 |  |
| rs9322676 | 5.47E-01 | 9.14E-01 | 0.598 | 0.5496 |  |
| rs1431213 | 5.21E-01 | 3.81E-01 | 1.367 | 0.17156 |  |
| rs1272565 | -1.41E-01 | 4.73E-01 | -0.298 | 0.7659 |  |
| rs287908 | -4.42E-01 | 7.63E-01 | -0.58 | 0.56214 |  |
| rs10499295 | -3.25E-01 | 6.24E-01 | -0.52 | 0.60305 |  |
| rs10499297 | 5.60E-01 | 7.77E-01 | 0.721 | 0.47074 |  |
| rs10499278 | 8.19E-01 | 8.79E-01 | 0.932 | 0.35139 |  |
| rs4132167 | 8.41E-01 | 1.32E+00 | 0.636 | 0.52458 |  |
| rs2865113 | 2.63E-01 | 2.14E+00 | 0.123 | 0.90205 |  |
| rs970761 | 7.80E-01 | 7.36E-01 | 1.059 | 0.28966 |  |
| rs10246631 | -5.77E-01 | 5.63E-01 | -1.024 | 0.30567 |  |
| rs7809475 | 2.62E-02 | 4.65E-01 | 0.056 | 0.95507 |  |
| rs3922333 | -1.29E-01 | 4.72E-01 | -0.272 | 0.78529 |  |
| rs1543239 | 3.54E-01 | 4.27E-01 | 0.828 | 0.40751 |  |
| rs10499810 | 2.14E-15 | 2.50E+00 | 0 | 1 |  |
| rs10487949 | 1.31E-01 | 7.86E-01 | 0.167 | 0.86736 |  |
| rs10499813 | -7.29E-01 | 6.48E-01 | -1.125 | 0.26052 |  |
| rs10263639 | 1.53E-01 | 4.58E-01 | 0.335 | 0.73786 |  |
| rs10503561 | 2.78E-01 | 3.23E-01 | 0.861 | 0.38949 |  |
| rs1823938 | 2.65E-01 | 8.94E-01 | 0.297 | 0.76673 |  |
| rs10503535 | -1.17E-01 | 4.52E-01 | -0.258 | 0.79644 |  |
| rs10504289 | 4.75E-01 | 4.82E-01 | 0.984 | 0.32507 |  |
| rs10504291 | 1.12E+00 | 2.05E+00 | 0.545 | 0.5857 |  |
| rs2261824 | 5.69E-01 | 4.23E-01 | 1.347 | 0.17797 |  |
| rs2319420 | 2.58E-01 | 6.75E-01 | 0.383 | 0.70195 |  |
| rs10491711 | -3.31E-01 | 5.74E-01 | -0.577 | 0.56393 |  |
| rs8210 | 2.61E-01 | 5.04E-01 | 0.518 | 0.60417 |  |
| rs1479238 | -2.11E-03 | 4.82E-01 | -0.004 | 0.99651 |  |
| rs5986238 | 1.42E-01 | 2.99E-01 | 0.476 | 0.6344 |  |
| rs763915 | 2.94E-15 | 2.50E+00 | 0 | 1 |  |
| rs727381 | 5.90E-01 | 5.11E-01 | 1.155 | 0.24815 |  |
| rs431207 | -1.20E-01 | 4.41E-01 | -0.273 | 0.78497 |  |
| rs6610546 | -3.94E-01 | 3.11E-01 | -1.265 | 0.20588 |  |
| rs10521971 | -1.97E-01 | 1.31E+00 | -0.15 | 0.88069 |  |
| rs2206338 | 4.38E-01 | 3.05E-01 | 1.435 | 0.15119 |  |
| rs5918214 | 3.24E-01 | 3.60E-01 | 0.9 | 0.3682 |  |
| rs4472657 | 2.48E-01 | 4.48E-01 | 0.553 | 0.57995 |  |
| rs945052 | -1.27E+00 | 1.55E+00 | -0.819 | 0.41266 |  |
| rs1891876 | 2.56E-15 | 2.50E+00 | 0 | 1 |  |
| rs10521531 | -2.78E+00 | 1.55E+00 | -1.794 | 0.07288 | . |
| rs4129094 | -4.53E-01 | 3.38E-01 | -1.339 | 0.18044 |  |
| rs10521535 | 1.27E+00 | 1.19E+00 | 1.064 | 0.28735 |  |
| rs10521533 | 6.59E-01 | 2.25E+00 | 0.293 | 0.7694 |  |
| rs7886027 | 6.59E-01 | 2.25E+00 | 0.293 | 0.7694 |  |
| rs10521757 | 0.00E+00 | 2.50E+00 | 0 | 1 |  |
| rs970666 | -1.83E-01 | 4.41E-01 | -0.414 | 0.67869 |  |

**Table S9. Intellectual Disability GPL2004 rs10493766 synergistic partners and scores**

| rsID | Estimate | Std. Error | z value | Pr(>\|z\|) | Sig. |
| --- | --- | --- | --- | --- | --- |
| rs233072 | 1.55E-01 | 8.14E-01 | 0.191 | 0.8488 |  |
| rs10493766 | -2.70E-01 | 4.54E-01 | -0.594 | 0.5527 |  |
| rs624860 | 1.94E-15 | 2.50E+00 | 0 | 1 |  |
| rs997251 | -8.11E-01 | 8.02E-01 | -1.012 | 0.3117 |  |
| rs10489511 | 3.33E-15 | 2.50E+00 | 0 | 1 |  |
| rs10518341 | 2.04E-15 | 2.50E+00 | 0 | 1 |  |
| rs10495470 | 8.83E-01 | 8.47E-01 | 1.043 | 0.2971 |  |
| rs10495460 | 1.72E-01 | 9.22E-01 | 0.187 | 0.8519 |  |
| rs10509120 | -5.42E-01 | 8.20E-01 | -0.661 | 0.5084 |  |
| rs2247808 | 1.84E+00 | 1.55E+00 | 1.186 | 0.2356 |  |
| rs2247812 | 5.34E-01 | 1.10E+00 | 0.485 | 0.6279 |  |
| rs3135739 | -1.83E+00 | 1.61E+00 | -1.136 | 0.256 |  |
| rs2981433 | -3.61E-01 | 1.79E+00 | -0.201 | 0.8407 |  |
| rs992176 | 6.93E-01 | 4.18E-01 | 1.659 | 0.0972 | . |
| rs10488695 | 8.90E-02 | 8.14E-01 | 0.109 | 0.913 |  |
| rs984895 | -3.73E-01 | 5.03E-01 | -0.741 | 0.4589 |  |
| rs4764011 | 5.05E-01 | 3.60E-01 | 1.401 | 0.1613 |  |
| rs722646 | 2.27E-15 | 2.50E+00 | 0 | 1 |  |
| rs10492134 | 5.19E-01 | 4.47E-01 | 1.162 | 0.2454 |  |
| rs10505892 | -5.93E-01 | 4.24E-01 | -1.398 | 0.162 |  |
| rs10505890 | 2.10E-01 | 1.56E+00 | 0.135 | 0.8928 |  |
| rs10505886 | 1.87E-15 | 2.50E+00 | 0 | 1 |  |
| rs4129990 | 2.10E-01 | 1.56E+00 | 0.135 | 0.8928 |  |
| rs7957614 | 3.70E-01 | 7.54E-01 | 0.491 | 0.6236 |  |
| rs10507027 | 6.68E-01 | 9.35E-01 | 0.715 | 0.4745 |  |
| rs7997094 | -1.49E+00 | 1.14E+00 | -1.307 | 0.1912 |  |
| rs10507360 | -1.61E-01 | 1.25E+00 | -0.129 | 0.8971 |  |
| rs10492426 | 1.26E+00 | 1.47E+00 | 0.858 | 0.3907 |  |
| rs9331441 | 2.19E-15 | 2.50E+00 | 0 | 1 |  |
| rs10492828 | 2.51E-15 | 2.50E+00 | 0 | 1 |  |
| rs844395 | -4.33E-01 | 4.61E-01 | -0.939 | 0.3476 |  |
| rs10492827 | -5.27E-01 | 5.99E-01 | -0.88 | 0.3789 |  |
| rs9319551 | -6.80E-01 | 5.18E-01 | -1.312 | 0.1894 |  |
| rs10514490 | 1.61E+00 | 1.35E+00 | 1.195 | 0.2319 |  |
| rs2009458 | -9.30E-01 | 4.14E-01 | -2.244 | 0.0248 | * |
| rs10514459 | -6.30E-01 | 7.09E-01 | -0.889 | 0.3742 |  |
| rs8090078 | 8.80E-01 | 1.05E+00 | 0.835 | 0.4039 |  |
| rs10496791 | 2.00E-15 | 2.50E+00 | 0 | 1 |  |
| rs10496789 | -1.05E+00 | 2.06E+00 | -0.507 | 0.612 |  |
| rs10497797 | -1.03E-01 | 1.98E+00 | -0.052 | 0.9586 |  |
| rs9305974 | -6.84E-01 | 8.23E-01 | -0.832 | 0.4056 |  |
| rs2826474 | -4.70E-01 | 5.64E-01 | -0.834 | 0.4045 |  |
| rs10482909 | -3.19E-01 | 1.65E+00 | -0.193 | 0.8466 |  |
| rs10483185 | -9.01E-01 | 9.88E-01 | -0.912 | 0.3618 |  |
| rs10483174 | -5.09E-01 | 1.05E+00 | -0.486 | 0.6273 |  |
| rs5999110 | -8.28E-01 | 4.78E-01 | -1.732 | 0.0833 | . |
| rs2413201 | -2.39E-01 | 6.20E-01 | -0.385 | 0.7 |  |
| rs5754819 | -2.28E-01 | 7.62E-01 | -0.299 | 0.765 |  |
| rs1508744 | 5.33E-01 | 4.55E-01 | 1.173 | 0.2409 |  |
| rs711623 | -1.17E+00 | 1.49E+00 | -0.785 | 0.4324 |  |
| rs7637204 | 1.42E-15 | 2.50E+00 | 0 | 1 |  |
| rs4016435 | -5.18E-01 | 8.00E-01 | -0.648 | 0.5172 |  |
| rs919095 | 2.48E-15 | 2.50E+00 | 0 | 1 |  |
| rs919096 | -2.72E-01 | 1.38E+00 | -0.198 | 0.8433 |  |
| rs10510711 | -8.77E-01 | 1.13E+00 | -0.774 | 0.4388 |  |
| rs9310179 | -9.25E-02 | 1.02E+00 | -0.091 | 0.9277 |  |
| rs9310178 | -9.25E-02 | 1.02E+00 | -0.091 | 0.9277 |  |
| rs1024889 | 7.99E-01 | 4.52E-01 | 1.765 | 0.0776 | . |
| rs162428 | 4.16E-01 | 4.96E-01 | 0.838 | 0.4023 |  |
| rs1507422 | 2.35E-15 | 2.50E+00 | 0 | 1 |  |
| rs17300673 | -8.28E-01 | 6.19E-01 | -1.338 | 0.181 |  |
| rs10514741 | -2.75E-01 | 8.72E-01 | -0.315 | 0.7524 |  |
| rs9309788 | -2.32E-01 | 8.49E-01 | -0.273 | 0.7849 |  |
| rs864380 | -4.43E-01 | 4.22E-01 | -1.05 | 0.2939 |  |
| rs6846301 | -1.92E-02 | 5.73E-01 | -0.034 | 0.9732 |  |
| rs9291201 | -9.97E-01 | 8.40E-01 | -1.186 | 0.2355 |  |
| rs6848363 | -5.74E-01 | 1.39E+00 | -0.414 | 0.6787 |  |
| rs580758 | -1.14E-01 | 4.87E-01 | -0.234 | 0.815 |  |
| rs10516508 | -3.15E-01 | 1.09E+00 | -0.29 | 0.7722 |  |
| rs10488863 | 0.00E+00 | 2.50E+00 | 0 | 1 |  |
| rs2866685 | 5.59E-01 | 4.09E-01 | 1.367 | 0.1718 |  |
| rs27759 | -2.05E-01 | 6.00E-01 | -0.341 | 0.733 |  |
| rs10513177 | 2.27E-15 | 2.50E+00 | 0 | 1 |  |
| rs7715916 | -7.76E-01 | 7.70E-01 | -1.009 | 0.3131 |  |
| rs3933494 | 1.27E+00 | 2.01E+00 | 0.633 | 0.5269 |  |
| rs10514321 | -4.30E-01 | 8.94E-01 | -0.482 | 0.6301 |  |
| rs10514309 | -4.21E-01 | 7.41E-01 | -0.568 | 0.5699 |  |
| rs10505857 | -3.59E-01 | 5.39E-01 | -0.667 | 0.5049 |  |
| rs9296469 | 6.07E-01 | 4.77E-01 | 1.272 | 0.2034 |  |
| rs10498763 | -4.38E-02 | 9.22E-01 | -0.047 | 0.9622 |  |
| rs681611 | 2.31E-15 | 2.50E+00 | 0 | 1 |  |
| rs2247215 | 1.90E-01 | 4.19E-01 | 0.452 | 0.6512 |  |
| rs10499033 | -1.10E+00 | 8.12E-01 | -1.352 | 0.1764 |  |
| rs10485272 | 2.34E-01 | 1.81E+00 | 0.129 | 0.8973 |  |
| rs10484475 | 3.88E-01 | 1.24E+00 | 0.314 | 0.7534 |  |
| rs10499294 | 2.38E-15 | 2.50E+00 | 0 | 1 |  |
| rs10485197 | 1.45E+00 | 1.48E+00 | 0.98 | 0.3271 |  |
| rs2236421 | -3.15E-03 | 6.30E-01 | -0.005 | 0.996 |  |
| rs917617 | 1.56E+00 | 8.15E-01 | 1.918 | 0.0551 | . |
| rs3911826 | -3.17E-01 | 2.13E+00 | -0.149 | 0.8815 |  |
| rs2204339 | 3.00E-01 | 8.45E-01 | 0.355 | 0.7225 |  |
| rs6460458 | 2.83E-01 | 3.85E-01 | 0.733 | 0.4634 |  |
| rs4718954 | -3.17E-01 | 2.13E+00 | -0.149 | 0.8815 |  |
| rs10230101 | 9.26E-01 | 1.37E+00 | 0.675 | 0.4995 |  |
| rs10486861 | 7.87E-02 | 8.53E-01 | 0.092 | 0.9266 |  |
| rs10486876 | -7.50E-01 | 9.09E-01 | -0.826 | 0.4091 |  |
| rs7002469 | 2.72E-01 | 3.91E-01 | 0.697 | 0.4858 |  |
| rs10503528 | -1.14E+00 | 6.87E-01 | -1.664 | 0.0961 | . |
| rs10503527 | -6.46E-01 | 1.16E+00 | -0.557 | 0.5772 |  |
| rs10503560 | -1.45E-01 | 6.37E-01 | -0.227 | 0.8204 |  |
| rs10503551 | -4.56E-01 | 8.55E-01 | -0.534 | 0.5934 |  |
| rs1511029 | 1.85E-15 | 2.50E+00 | 0 | 1 |  |
| rs10504305 | -6.72E-02 | 1.37E+00 | -0.049 | 0.961 |  |
| rs3122691 | -6.79E-01 | 5.06E-01 | -1.342 | 0.1795 |  |
| rs2326457 | 4.61E-01 | 1.37E+00 | 0.336 | 0.7369 |  |
| rs10504296 | 1.97E-15 | 2.50E+00 | 0 | 1 |  |
| rs4736236 | -1.57E+00 | 1.36E+00 | -1.157 | 0.2472 |  |
| rs10511435 | 9.30E-01 | 8.45E-01 | 1.1 | 0.2712 |  |
| rs759656 | -7.07E-01 | 4.02E-01 | -1.761 | 0.0782 | . |
| rs10491718 | 5.45E-16 | 2.50E+00 | 0 | 1 |  |
| rs7022827 | -3.29E-01 | 5.31E-01 | -0.619 | 0.5357 |  |
| rs2468705 | -5.85E-01 | 4.67E-01 | -1.255 | 0.2096 |  |
| rs5973759 | 2.06E-01 | 4.06E-01 | 0.506 | 0.6126 |  |
| rs10521965 | -5.13E-02 | 3.41E-01 | -0.15 | 0.8805 |  |
| rs10521906 | 2.10E-15 | 2.50E+00 | 0 | 1 |  |
| rs197015 | -1.86E-01 | 3.34E-01 | -0.557 | 0.5775 |  |
| rs10521966 | -7.07E-02 | 4.31E-01 | -0.164 | 0.8698 |  |
| rs10521968 | 2.03E-15 | 2.50E+00 | 0 | 1 |  |
| rs5937962 | 6.41E-01 | 2.04E+00 | 0.315 | 0.7531 |  |
| rs10521424 | 1.88E-15 | 2.50E+00 | 0 | 1 |  |
| rs4481736 | 6.66E-02 | 4.92E-01 | 0.135 | 0.8924 |  |
| rs5952044 | -1.20E+00 | 1.55E+00 | -0.778 | 0.4368 |  |

Signif. codes: 0 ‘***’ 0.001 ‘**’ 0.01 ‘*’ 0.05 ‘.’ 0.1 ‘ ’ 1


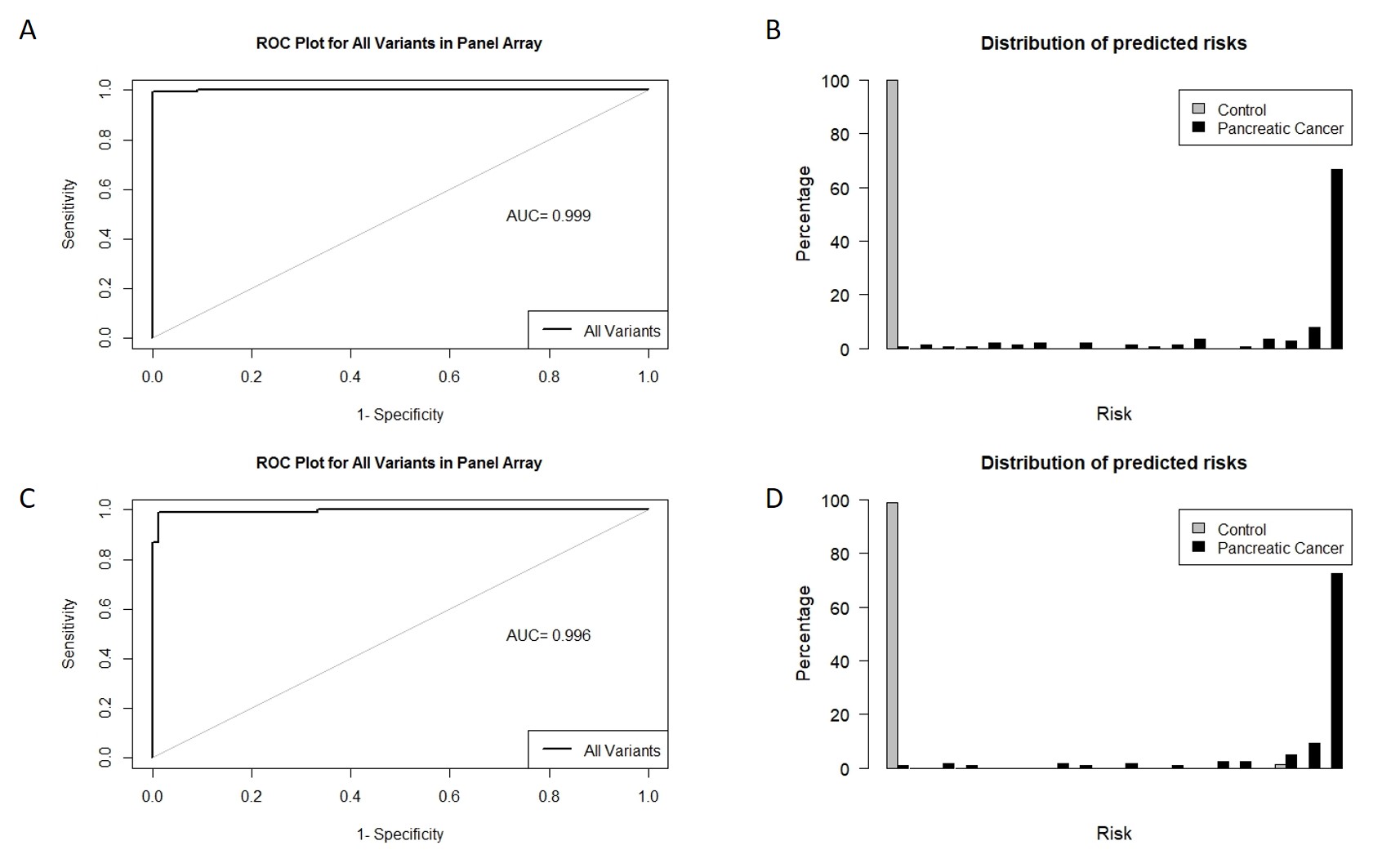


**Figure S1. Risk assessment for Validation cohorts** (A) ROC curve of affected (with outcome) versus unaffected (without outcome) distribution for the GSE8055 validation cohort of 141 patients with pancreatic cancer and 89 controls for 928 variants known to be associated with pancreatic cancer (B) Distribution of disease control pancreatic cancer cohort GSE8055. (C) ROC curve of affected (with outcome) versus unaffected (without outcome) distribution for the GSE8054 validation cohort of 121 patients with pancreatic cancer and 87 controls for 1189 variants known to be associated with pancreatic cancer (D) Distribution of disease control pancreatic cancer cohort GSE8054.

**Figure S2. Incremental removal of all variants.** The plot shows the AUC for models 1 and 2 (see Material and Methods). Model 1 shows the AUC score for the analysis of top variant rs6905948 from the GSE8055 dataset including all its synergistic partners (blue line). The orange line shows the incremental removal of each variant depicting the drop in prediction accuracy as shown by the AUC score. The greatest drop in AUC scores (0.854 to 0.787) occurs when the rs6905948 variant is removed from the analysis.


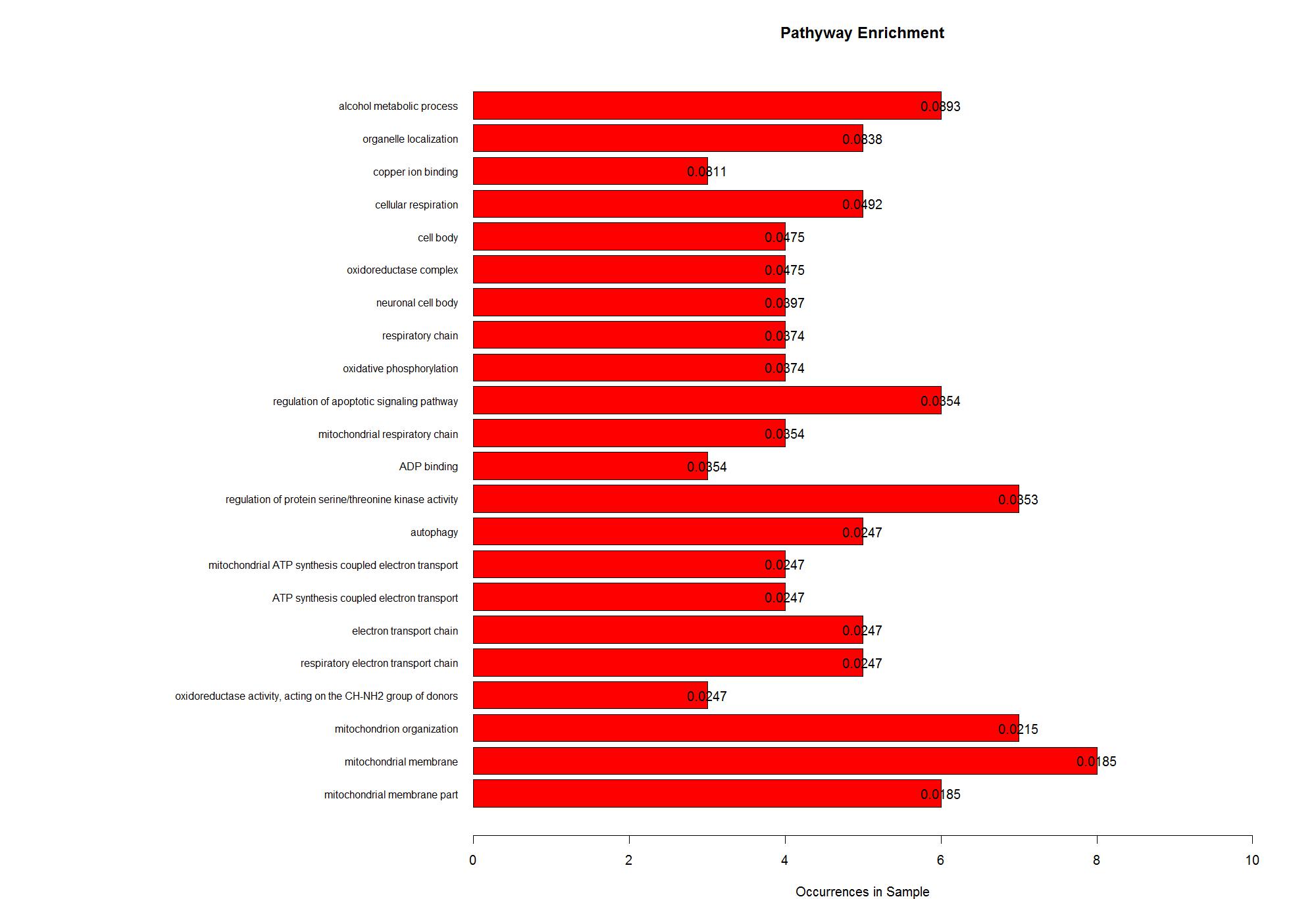


**Pathway Enrichment**

**Figure S3. Pathway Enrichment analysis of synergistic variant genes obtained from** Parkinson's **subnetwork.** Parkinson's disease co-expression network obtained by selecting the genes for the top variant rs3172404 and its synergistic partners, The vertical axis shows the 22 most significantly enriched molecular functions, cellular components and biological processes as found in GO. The red bars represent gene occurrence in samples and are further labelled with the q-value to highlight significance. The plot shows significant enrichment in mitochondrial-related and energy-producing functional themes that are key molecular players in neurodenerative disorders.


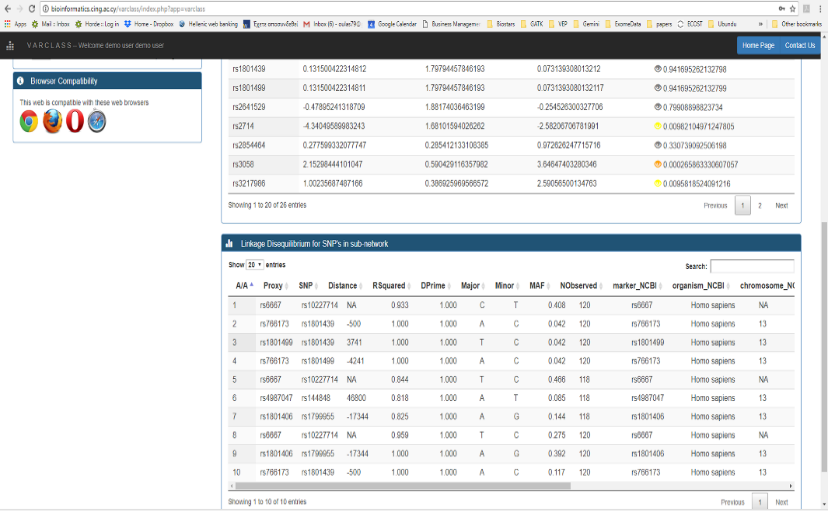

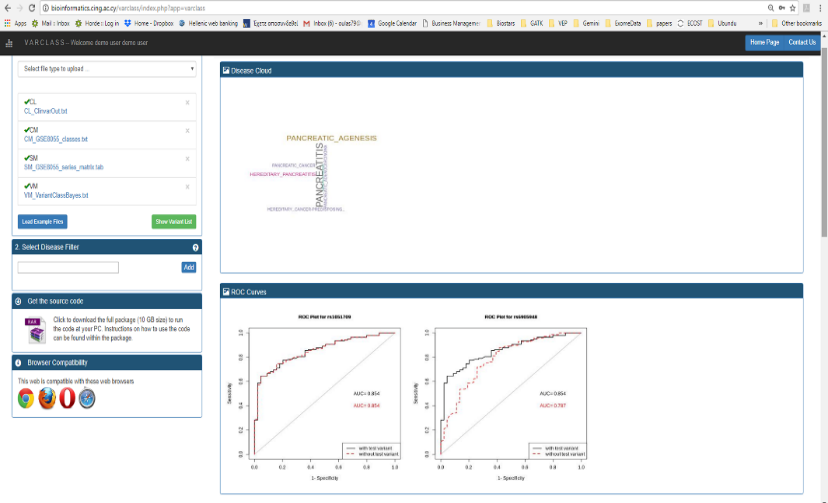

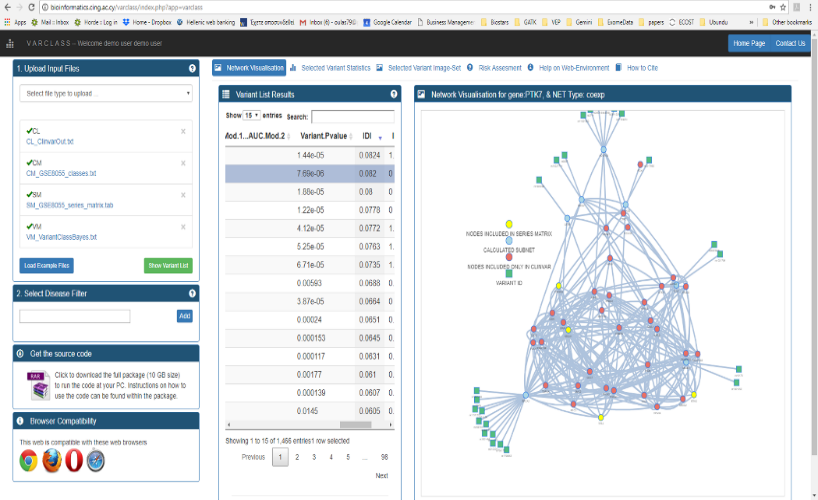

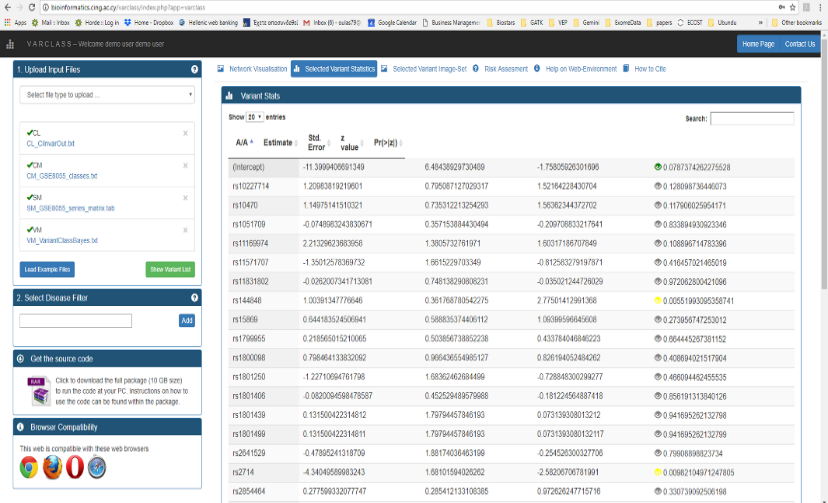

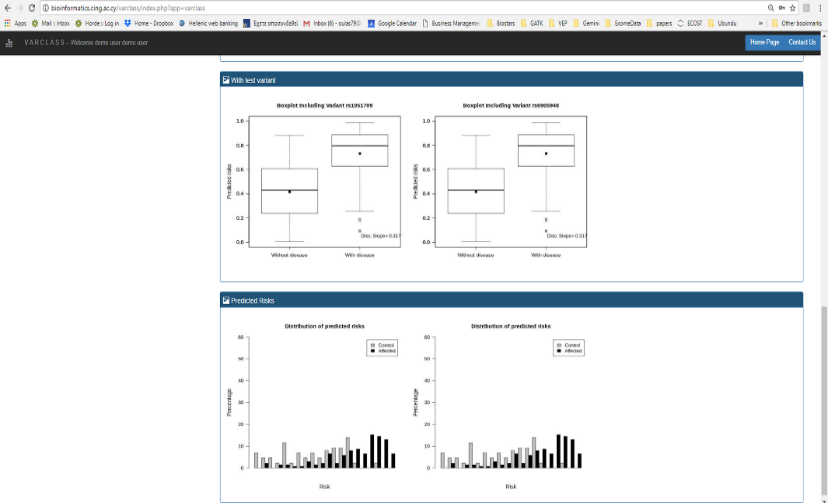

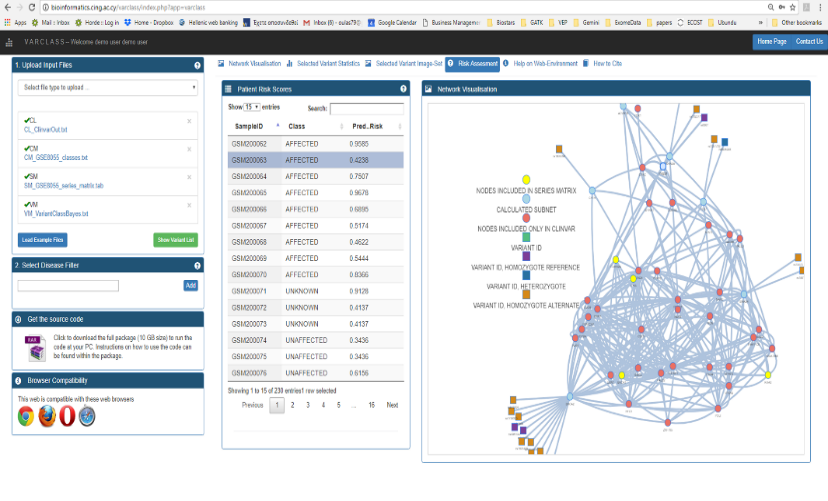


A

B

C

D

E

F

**Figure S4. The VarClass Web Interface** – Snapshots from the web version of VarClass (<http://www>). (A) Selecting top Scoring Variants (i.e. PTK7 gene and variant) and visualization of subnetwork including neighbouring genes and synergistic variant partners. (B) Statistics of Variants in Subnet including AUC score for models 1 (including unknown variant) and 2 (without unknown variant), IDI values for variants and p-values for variant coefficients from linear regression model. (C) Linkage Disequilibrium for subnetwork variants derived from SNP IDs. (D) Disease Classification and ROC curves from ClinVar and risk assessment using model 1 and model 2. (E) Boxplots and distribution plots for models 1 and 2 for outcome versus no outcome, (F) Risk score for individual samples in the GWAS or WES dataset and personalized/individualized networks showing genotypes for specific selected sample.


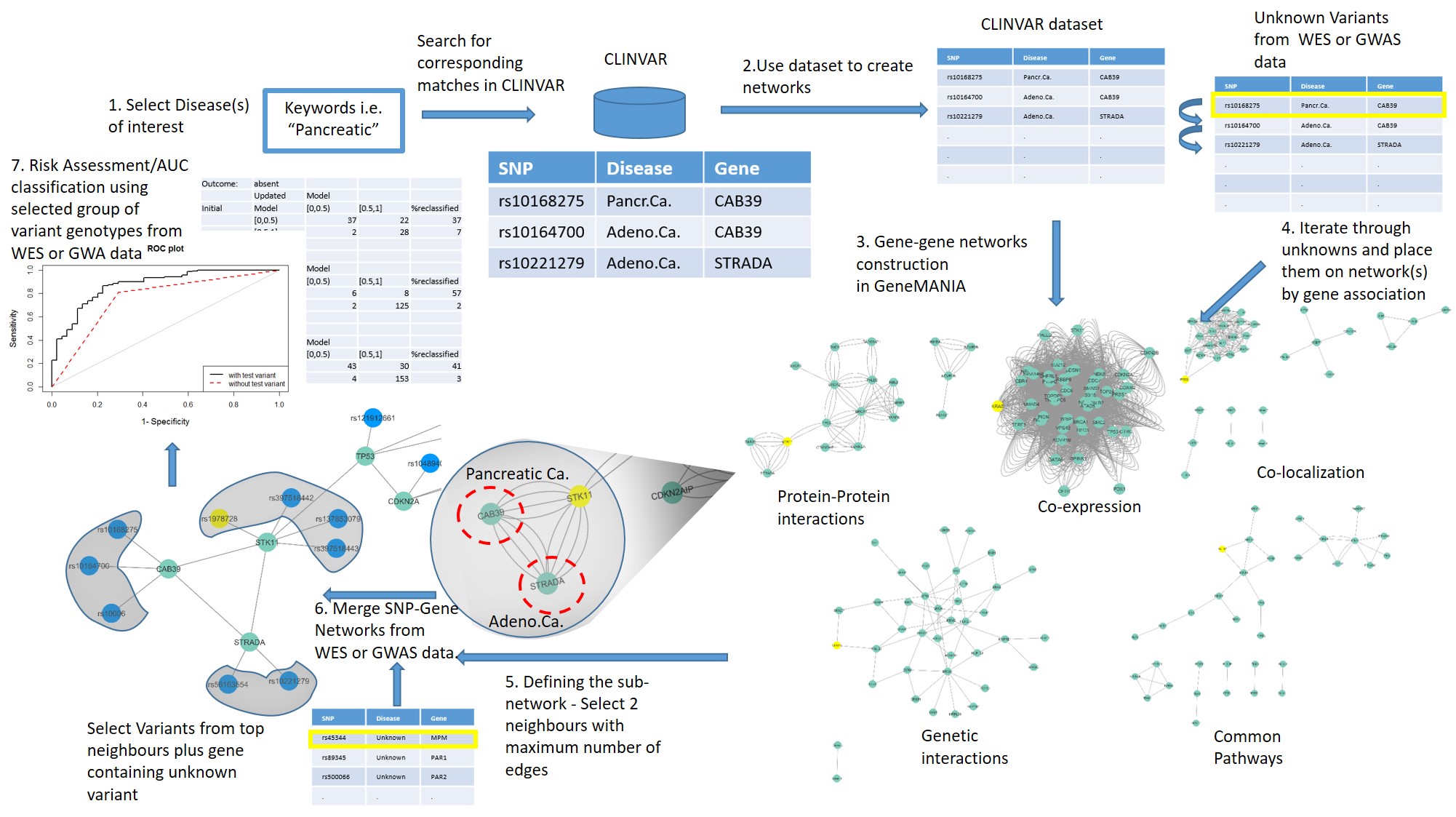


**Figure S5. Graphical abstract of VarClass Pipeline**

*Step 1: Selecting disease direction/profile -* The VarClass approach requires a general disease direction to initiate the pipeline. When using the web tool, the user can query ClinVar which is stored locally on our servers as a MySQL database. Querying is performed by adding keywords of interests in order to extract the relevant information. This allows for a specific direction to be chosen and aids in the construction of disease-related profile that can be targeted to specific diseases of interest (e.g. Pancreatic cancer) or allowing for a broader profile encapsulating other types of cancers (i.e. shifting to the family of adenocarcinomas – which describes cancerous tumours that can occur in several parts of the body such as neoplasia of epithelial tissue that has glandular origin, glandular characteristics, or both, for example stomach or prostate cancer).

*Step 2. Extracting relevant information from ClinVar -* The relevant information by simple SQL querying, is extracted from ClinVar. This includes the variant id (e.g. rs2229992), the gene that harbours the underlying variation (e.g. APC) and also the clinical outcome name associated with the given entry (e.g. Colorectal Cancer). This compiled information creates the specific disease direction/profile described in step 1.

*Step 3. Network Construction -* The gene information (gene symbols) extracted from all entries associated with the disease profile (as defined in steps 1 and 2) are used to construct the backbone of the gene-to-gene network(s). This is achieved by using the gene symbols as input to GeneMANIA in order to construct 5 different types of biologically relevant, disease-specific networks. More details are described in the “*Network Construction using geneMANIA”* section below. These networks are then used for later stages of the pipeline.

*Step 4. Placing unknown variants on the networks* – unknown variants are placed iteratively on all five networks by means of gene association. This is achieved by using GeneMANIA to obtain relationships between the gene harbouring the VUS and other genes in the disease specific networks. Hence, each VUS is now incorporated into a biological, evidence-based network backbone underlining the disease of interest.

*Step 5. Defining the sub-network of informative variants* – Firstly, this step involves the selection for the top 2 neighbours (see main text *Validation of VarClass* for details) of the gene harbouring the VUS. These neighbours are next used for *prediction of clinical outcome for VUS* (note - this prediction is only informative if more than two diseases have been incorporated into the disease profile). VarClass looks at the clinical outcome of these gene neighbours of the gene harbouring the VUS and assigns a clinical outcome to the VUS based on these. Running validation analyses (see main text *Validation of VarClass* for details) shows that optimal results are achieved when top 2 neighbours (nodes) by highest number of interactions (edges) are selected (keeping in mind that if one or more neighbours have the same number of interactions, they are also included in the top neighbours). These mostly include neighbours that are supported by multiple data sources in GeneMANIA (i.e. protein-protein interactions supported by 2 sources, e.g. BioGRID and PathwayCommons, will be joined by 2 edges). Secondly, the subnetwork is further expanded by selecting the 2^nd^ order neighbours (i.e. neighbours of the top 2 neighbouring genes), hence adding even more informative genes for the next processing steps of the analysis pipeline.

*Step 6. Extract variant IDs from real data* – this next step involves the use of real GWAS/WES data and incorporating information from these datasets into the network(s) while at the same time extrapolating useful disease specific and biologically meaningful information from the network(s). This is performed by adding all the variants from the GWAS/WES datasets to their corresponding genes present in the selected subnetwork(s).

*Step 7. Using variants derived from sub-networks for risk prediction* – The variants obtained from the sub-network (keeping in mind that some of these may be also VUS) are used in the risk model construction using the genotypes from all disease and control samples in the GWAS/WES study. Two types of risk models are generated. Namely, Model 1 - which contains all the sample genotypes from the variants found in the subnetwork and Model 2 - a second model that contains all genotypes minus the genotypes of the VUS that is under investigation at that given iteration. This allows for two risk predictions and hence entails the assessment of two types of models for their ability to classify samples with respect to accuracy in risk prediction and disease outcome. To avoid overfitting, 5-fold cross-validation procedure is adopted during classification. Measures used for model assessment include ROC/AUC, NRI, and IDI (for details see section for ***Risk score prediction using Linear Logistic Regression Analysis and risk model construction***). The difference in AUC, NRI and IDI between the two models provides a means of assessing the contribution of the VUS under investigation. It is anticipated that VUS that contribute towards accurate risk prediction should lower model performance if removed. However, minor changes, although highly significant, are expected as it is the synergy of all variants that allow for good quality in model performance and not the effect of any variant alone.

Colour coding: The yellow rectangles (steps 2-3 and steps 5-6) highlight the variant (VUS) that is under investigation for this specific iteration of the pipeline. The green nodes (shown in steps 3-6) represent genes found in gene-gene networks. The yellow nodes (steps 3-6) represent the gene/variant been analysed in this VarClass iteration. The blue nodes depict additional informative variants assigned to the top neighbouring genes found in sub-network are extracted from WES or GWAS (step 6).
